# Online Parenting Programs for Children’s Behavioral and Emotional Problems: a Network Meta-Analysis

**DOI:** 10.1007/s11121-024-01735-1

**Published:** 2024-10-13

**Authors:** Ana Catarina Canário, Rita Pinto, Marco Silva-Martins, Karen Rienks, Burcu Kömürcü Akik, Koraljka Modić Stanke, Oana David, Rukiye Kızıltepe, G. J. Melendez-Torres, Therdpong Thongseiratch, Patty Leijten

**Affiliations:** 1 Faculty of Psychology and Education Sciences, University of Porto, Rua Alfredo Allen S/N, 4200-135 Porto, Portugal; 2 University of Amsterdam, Amsterdam, Netherlands; 3 Ankara University, Ankara, Turkey; 4 University of Zagreb, Zagreb, Croatia; 5 Babeş-Bolyai University, Cluj-Napoca-Napoca, Romania; 6 Pamukkale University, Denizli, Turkey; 7 University of Exeter, Exeter, England; 8 Prince of Songkla University, Hat Yai, Thailand

**Keywords:** Online parenting programs, Child behavioral problems, Child emotional problems, Parents’ parenting practices, Parents’ mental health, Meta-analysis

## Abstract

Online parenting programs to support parents of children with behavioral problems and emotional problems have become widely available in recent years. Research has consistently shown their positive effects on child development, parents’ adaptive parenting practices, and parents’ mental health. However, knowledge is lacking on which type of content is more suitable to be delivered online. Our work addresses this knowledge gap by conducting traditional and network meta-analyses to improve our understanding of (1) how effective online parenting programs are to improve children’s behavior and emotional problems, and (2) what clusters of components are most likely to yield the strongest effects. Following the PROSPERO preregistration, we systematically searched PsycINFO, MEDLINE, Web of Science, and Cochrane. Of the 8292 records retrieved, 28 records on 27 randomized controlled trials (*N* = 5,312) met the inclusion criteria. Results show moderate effect sizes of online parenting programs on reduced child behavioral and emotional problems, parents’ ineffective parenting practices, and parents’ mental health problems. Online programs adopting a learning theory perspective, either with or without additional parental self-care and parents as therapist approaches, are most likely to yield the strongest effects on child behavioral problems. Online programs adopting a learning theory perspective, parental self-care and parents as therapist approaches, with or without additional relationship perspectives, are most likely to yield the strongest effects on child emotional problems. Online parenting programs seem promising tools for improving child behavioral and emotional problems. Future research should identify the circumstances that allow parents and children to benefit more from specific components in these programs.

**Supplementary Information:**

The online version contains supplementary material available at 10.1007/s11121-024-01735-1.

## Introduction

Online delivery of parenting programs has significantly increased since the COVID-19 pandemic (McAloon & de la Poer Beresford, 2023; Sullivan et al., 2021). Studies consistently show that online parenting programs can successfully improve parents’ understanding of child development, self-efficacy, adaptive parenting behavior, and children’s behavioral and emotional problems (David et al., 2023; Opie et al., 2024; Spencer et al., 2020). Little is known, however, about what parenting program content is most suitable to be delivered online. The literature lacks well-powered evaluations of different types of online programs and the components that have more generalized or unique effects on children’s behavioral and emotional problems. The current study addresses this knowledge gap by conducting traditional and network meta-analysis to answer the research questions: How effective are online parenting programs to improve children’s behavioral and emotional problems? What clusters of components are most likely to yield the strongest effects?

### Parenting Programs for Children’s Behavioral and Emotional Problems

Parenting programs are structured interventions that aim to improve parenting practices and subsequently reduce children’s behavioral and emotional difficulties (Prinz, 2019). These programs are known to have wider effects also on parent–child relationship quality, parenting practices, and family well-being and reduce violence against children (Backhaus et al., 2023; Jervis et al., 2023; Thomas & Zimmer-Gembeck, 2007). These positive effects are robust across a variety of populations, including parents with mental health problems (Jones et al., 2017), parents involved with child protective services (Leclair Mallette et al., 2021), and parents of children with diagnosis for mental disorders (Jolstedt et al. 2018). Parenting programs’ effects on children’s behavioral and emotional problems are often examined in separate studies (e.g., Schwartz et al., 2019; Tehrani et al., 2023). Yet, behavioral and emotional problems often coexist (Caspi & Moffitt, 2018), and thus, it is important to see if the same program content benefits both problems, and to what extent.

### Online Parenting Programs: Evidence for Their Effects and Components

Many programs that were initially developed to be delivered in person are now also delivered online. This happened first as a consequence of the technological progress made in the last decades and, more recently, due to the COVID-19 pandemic (Sullivan et al., 2021). Frequently used types of online parenting support include programs delivered through websites (e.g., Day & Sanders, 2018), apps (David et al., 2024), and videoconference platforms (e.g., Canário et al., 2021). These programs can be delivered with guidance from experts or peers, without guidance (i.e., self-directed), or in combination. Their advantages, relative to in-person delivery, include more flexible scheduling and enhanced accessibility (Canário et al., 2022).

Several meta-analyses have addressed the effects of online parenting programs on child and family well-being. Results reporting small to moderate effect sizes immediately postintervention and at late follow-up assessments suggest that the programs are effective in reducing child and adolescent behavioral and emotional problems (David et al., 2023; Florean et al., 2020; Spencer et al., 2020; Thongseiratch et al., 2020). Similarly, results suggest that the programs are effective in improving parents’ parenting practices, self-efficacy, and behavior; reducing parents’ mental health problems; and improving parents’ conflict, parent–child relationship quality, and parents’ social support across parents of children and adolescents under the age of 18 (Florean et al., 2020; Opie et al., 2024; Spencer et al., 2020; Thongseiratch et al., 2020).

Further research is now needed to deepen our understanding of how the content of online parenting programs contributes to its effects. Program content is often studied in terms of program components: the specific parenting strategies taught within the program (Kaminski et al., 2008; Leijten et al., 2022). Because these techniques cluster within programs based on their theoretical approach (e.g., learning theory versus relational perspectives) (Melendez-Torres et al., 2015), we study content in terms of the clusters of components that reflect a program’s theoretical approach. More specifically, we examine whether the presence (versus absence) of specific clusters of components is associated with program effects and whether we can establish a ranking of the most effective combinations of clusters of components. The advantage of such a ranking is that it provides insights into which parenting program content is most likely to yield significant benefits for families.

Research has shown that in-person parenting programs combining techniques to improve positive parent–child interactions and emotional communication skills, and teach parents to use time out, highlighting the importance of parenting consistency and requiring parents to practice new skills with their children, led to greater program effectiveness on child behavior problems (Kaminski et al., 2008). More recently, research addressed the effects of clusters of components under the theoretical approaches of behavior management, parental self-management, relationship enhancement, and psychoeducation. Results showed that in-person parenting programs that included a behavior management approach, either without any additional approaches or in combination with parental self-management or relationship enhancement, had the greatest chance of being the most effective in reducing children’s behavioral problems (Leijten et al., 2022; Tehrani et al., 2023) and emotional problems (Costantini et al., 2023; Kjøbli et al., 2023).

Few studies have addressed the role of components in online parenting programs. McAloon and de la Poer Beresford’s (2023) systematic review suggests that being specific about applying contingent reinforcement of desirable or undesirable behavior seems to be related to decreasing child behavior problems. Thongseiratch et al. (2020) identified clusters of components that effectively contributed to the reduction of children’s behavioral and emotional problems. These included engaging in child-led play, parental self-emotion regulation, and also providing facilitator feedback and sending parents reminders to work on the program. However, research to date has not identified whether the same clusters of components are less or more effective for child behavioral and child emotional problems, or whether each outcome requires different content.

### The Current Study

To address this knowledge gap and deepen our understanding on whether all the programs’ contents can be equally suitable for online interventions, the current study estimates: (1) the effects of online parenting programs on child behavioral and emotional problems, parents’ ineffective parenting practices and mental health problems and (2) how different clusters of components affect child behavioral and emotional problems.

## Methods

### Preregistration and Reporting Guidelines

This systematic review was preregistered on PROSPERO (CRD42022354393) and follows PRISMA guidelines, including the extension statement for reporting systematic reviews incorporating network meta-analysis (Hutton et al., 2015; Moher et al., 2009; Page et al., 2021). We completed the PRISMA 27-item checklist and present it in Online Resource 1.

### Eligibility Criteria

Inclusion and exclusion criteria were defined in terms of Participants, Interventions, Comparisons, Outcomes, and Study designs (PICOS; Moher et al., 2009) based on the criteria defined by Thongseiratch et al. (2020). In terms of participants, eligible studies had a mean child age of 2 to 12 years old. To protect against inadvertent bias from specific populations, we excluded studies targeting parents of children in foster and residential care, parents of children with autism, and parents of children with severe disabilities or illnesses. In terms of intervention, eligible studies evaluated a parenting program where more than 50% of the overall program was delivered online and targeted parenting. Regarding the comparators, eligible studies compared the online parenting program to a passive or relatively passive control condition: against a waitlist, no or minimal intervention control condition. We excluded studies that compared the effects of an online parenting program to an in-person comparator, because these studies answer a different question: whether the delivery format (rather than the content) makes a difference for program effectiveness. Findings regarding the non-inferiority of online programs have recently been published elsewhere (Leijten et al., 2024). In terms of outcome, eligible studies included at least one of the following outcomes at any assessment postintervention: children’s behavior or emotional problems, parenting practices, or parent mental health problems. In terms of study design, eligible studies used randomized controlled trials.

We only included reports in English and published in peer-reviewed journals. Unpublished studies were not sought, because these were already excluded in the original review by Thongseiratch et al. (2020). We acknowledge that only including published studies may represent a bias in favor of significant results and therefore evaluated the risk of publication bias as part of our analyses.

### Information Sources, Search Strategy, and Selection Process

We updated the systematic literature search by Thongseiratch et al. (2020). We systematically searched PsycINFO, MEDLINE, Web of Science, and Cochrane for randomized controlled evaluations of online parenting programs targeting children’s behavior and emotional problems. Searches were run in May 2022 and updated in September 2023. We used keywords relating to (i) parenting (e.g., mother, father, family), (ii) support (e.g., intervention, program, coaching), and (iii) online delivery (e.g., digital, internet, e-health). Finally, we examined the reference lists of relevant systematic reviews and identified primary studies. Online Resource 2 includes our full search string. Titles and abstracts of 10% of the retrieved reports were screened in Rayyan (Ouzzani et al., 2016), independently by ACC and PL to identify potentially eligible studies (97% overlap; disagreements resolved through discussion). The full-text articles of these potentially eligible studies were independently assessed for inclusion and exclusion criteria by ACC and BKA (88% overlap; disagreements resolved through discussion).

### Data Collection Process and Items

For each study, we extracted information regarding (i) general study characteristics (e.g., year of publication, whether the trial was preregistered); (ii) program characteristics (e.g., name, type of delivery, prevention level); (iii) sample characteristics (e.g., children’s age, parental socioeconomic status); and (iv) standardized mean differences between conditions (i.e., sample size, means, and standard deviations for each outcome) for four outcome categories: child behavioral problems (i.e., symptoms of externalizing problems or behavior disorders), child emotional problems (i.e., symptoms of internalizing problems, anxiety or depression), parents’ ineffective parenting practices (i.e., parenting behavior known to predict child behavior or emotional problems; parenting behavior known to predict child mental health were included and their standardized mean difference reversed), and parents’ mental health problems (i.e., symptoms of stress, anxiety or depression). All data items were coded by ACC, and MSM, KMS, OD and RK coded the data items for 25% of the studies, each with adequate reliability (70 to 100% agreement; mean per item 87%).

Two authors, RP and KR, coded for the presence or absence of components under the following theoretical approaches: (i) psychoeducation, including knowledge transfer contents; (ii) relationship perspectives, including positive activities/involvement, child-led activities, mind-mindedness, or empathy; (iii) learning theory perspectives, including positive reinforcement, or non-violent disciplining techniques; (iv) proactive parenting, including direct commands, clear limits, or monitoring; (v) parental self-care, including parental stress reduction, parental emotion regulation, parental problem-solving skills, or parental partner support; and (vi) parents as therapist of their children, including parents actively teaching their children emotion regulation skills, problem-solving skills, social skills, or exposure/reducing avoidance. Further details on the definition and operationalization of each theoretical approach are provided in Table S1 (see Online Resource 3). We found adequate reliability (70% to 82% agreement; mean per item 74%) in the codification of the data items regarding the components’ contents.

### Effect Measures and Synthesis Methods

We used standardized mean difference as our effect size, calculated by subtracting postintervention means in the control condition from postintervention means in the intervention condition, divided by the pooled postintervention standard deviation. Standardized mean differences are expressed as Cohen’s *d* and reflect the number of standard deviations of decrease in the outcome of the intervention relative to the control condition.

First, we evaluated the effects of the online parenting programs for each outcome using robust variance estimation (Hedges et al., 2010) in Stata (v.16; StataCorp, 2019). Robust variance estimation takes the clustered nature of the data, with effect sizes nested within studies, into account, assuming a within-study correlation of effect sizes for the same outcome of 0.80.

We then performed network meta-analysis to compare the effects of each cluster of components on child behavioral problems and child emotional problems in two separate models in RStudio (RStudio Team, 2020), using the packages metafor (Viechtbauer, 2010) and netmeta (Schwarzer et al., 2015). The scripts used to run the network meta-analyses are provided in Online Resource 4. To estimate tau-squared, we used a restricted maximum likelihood estimator. For the clusters of components, we included the following theoretical approaches: relationship perspectives, learning theory perspectives, proactive parenting, parental self-care, and parents as therapist. We excluded psychoeducation as this was the most frequent component in the programs included in the review and would integrate almost all clusters. To deal with the multiple results when performing the network meta-analysis, we calculated a synthetic effect size for each comparison using *r* = 0.50. We then used ranking metrics to answer our second research question, determining which clusters of components were most likely to yield the strongest effects. The different clusters of components were assessed through relative rankings, comparing them with the control group on the variables of interest, children’s behavioral and emotional problems. The *p*-scores capture the position of clusters of components relative to others and the certainty of that position. Values range from 0 to 1, with higher values reflecting a higher position on the ranking. The *p* values cannot be interpreted in any absolute way.

### Risk of Bias and Publication Bias Assessment

We assessed study risk of bias using the Cochrane risk of bias tool 2.0 (Sterne et al., 2019). For each numerical data item collected, we performed a risk of bias assessment addressing the following domains: bias arising from the randomization process, bias due to deviations from intended interventions, bias due to missing outcome data, bias in measurement of the outcome, and bias in selection of the reported result. Results are presented in terms of low risk, some concerns, and high risk. The risk of bias plots was generated using the tool robvis (McGuinness & Higgins, 2021). We assessed publication bias through visual inspection of funnel plots for children’s behavioral and emotional problems, parent’s ineffective parenting practices, and mental health problems, and by testing whether the variance of effect sizes predicted effect size. A standard assumption of Egger’s test and trim-and-fill tests is the independence of effect sizes. Because we included all relevant effect sizes from each study, these tests were not applicable.

## Results

### Qualitative Synthesis

#### Study Selection and Characteristics

**Fig. 1:**
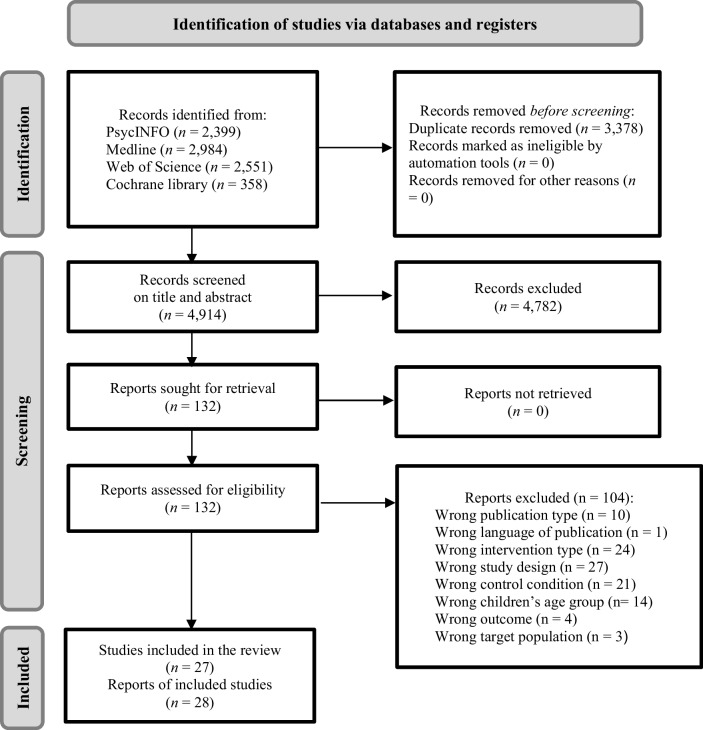
Flow chart of included reports in the systematic review

Our systematic literature search yielded 8292 records (Fig. 1). Twenty-seven studies of online parenting programs with a total of 5,312 participants, reported in 28 records, met the inclusion criteria (Table 1). Most records (*n* = 25, 89.3%) were published in the last decade; five trials evaluated the effects of online parenting programs during the COVID-19 pandemic (Chu et al., 2022; Comer et al., 2021; Palmer et al., 2023; Sung et al., 2021; Tuntipuchitanon et al., 2022). Most were conducted in the United States (*n* = 10, 37.0%), Europe (*n* = 7, 25.9%), or Australia (*n* = 6, 22.2%). Most trials had two arms, comparing one online parenting program to one control condition, except for five trials with three arms, comparing the online parenting program to both an active comparator and a control condition (Day & Sanders, 2018; de Jong et al., 2023; Khanna et al., 2017; Morgan et al., 2017; Tomlinson et al., 2023). In these trials, we extracted information regarding the comparison of the online parenting program to the control condition. We did not include the information regarding active comparators.

**Table 1:** Studies included in the review and their main characteristics

Study	Country	Control condition	Online program	Online delivery method	Prevention level	*N*	% Girls	Child age (*min–max* or *M*)	Outcome
Baker et al. (2017)	Australia	Waitlist	Triple P Online Brief	Website	Indicated	200	45.0	2 − 9 yrs	Child behavior problems (*ECBI*, *CAPES*); Child emotional problems (*CAPES*); Parents’ ineffective parenting practices (*PS*); Parents’ mental health problems (*DASS*)
Breitenstein et al. (2021)	USA	No or minimal intervention	ezParent Program	App	Universal	287	49.5	2 − 5 yrs	Child behavior problems (*ECBI,* *SDQ*); Child emotional problems (*SDQ*); Parents’ ineffective parenting practices (*PQ*, *PARYC*); Parents’ mental health problems (*PSI*)
Breitenstein et al. (2016)	USA	No or minimal intervention	ezParent Program	App	Universal	79	57.0	2 − 5 yrs	Child behavior problems (*ECBI*); Parents’ ineffective parenting practices (*PQ*); Parents’ mental health problems (*PSI*)
Chu et al. (2022)	China	Waitlist	Group Executive Functioning and Online Parent Training	Videoconference	Indicated	145	24.8	6 − 8 yrs	Child behavior problems (*SNAP-IV*); Parents’ mental health problems (*PSI*)
Comer et al. (2021)	USA	Waitlist	iCALM Telehealth Program	Videoconference	Indicated	40	73.0	3 − 8 yrs	Child emotional problems (*CBCL*); Parents’ mental health problems (*DASS*)
Day & Sanders (2018)	Australia	Waitlist	Triple P Online	Website	Selective	183	53.5	3.5 yrs	Child behavior problems (*ECBI*); Parents’ ineffective parenting practices (*PS*); Parents’ mental health problems (*DASS*)
DeGarmo & Jones (2019)	USA	Waitlist	Fathering Through Change	Website	Selective	426	44.0	4 − 12 yrs	Child behavior problems (*ECBI);* Parents’ ineffective parenting practices (*PPI*)
De Jong et al. (2023)	Netherlands	Waitlist	Self-Help Parenting Program	Website	Selective	73	27.3	8.17 yrs	Child behavior problems (*ECBI,* *EMA-EB*)
Donovan & March (2014)	Australia	Waitlist	Modified version of the BRAVE-ONLINE	Website	Indicated	52	53.9	3 − 6 yrs	Child emotional problems (*PAS, CBCL*)
DuPaul et al. (2018)	USA	Waitlist	Online Behavioral Parent Training	Website	Indicated	31	36.2	3 − 5 yrs	Child behavior problems (*CERS*); Parents’ mental health problems (*PSI*)
Ehrensaft et al. (2016)	USA	Waitlist	Triple P Online	Website	Selective	52	NR	2 − 6 yrs	Parents' ineffective parenting practices (*PS*); Parents’ mental health problems (*PSI*)
Enebrink et al. (2012)	Sweden	Waitlist	Internet-based Parent Management Training	Website	Indicated	104	42.0	3 − 12 yrs	Child behavior problems (*ECBI*, *SDQ*); Child emotional problems (*SDQ*); Parents’ ineffective parenting practices (*PPI*)
Farris et al. (2013)	USA	No or minimal intervention	Adventures in Parenting	Website	Universal	70	53.0	2 − 3 yrs	Mothers’ mental health problems (*SCL-90*)
Franke et al. (2020)	New Zealand	Waitlist	Triple P Online	Website	Indicated	53	28.3	3 − 4 yrs	Child behavior problems (*CERS,* *SDQ*); Parents’ ineffective parenting practices (*PS*, *PSDQ*); Parents’ mental health problems (*DASS*)
Guedes et al. (2023)	Portugal	Waitlist	Turtle Program	Videoconference	Selective	40	55.0	3 − 5 yrs	Child emotional problems (*PAS*); Parents’ ineffective parenting practices (*CRPRQ*)
Hutchings et al. (2023)	UK	Waitlist	Confident Parent Internet Guide program	Website	Universal	56	60.0	3 − 8 yrs	Child behavior problems (*ECBI*); Parents’ ineffective parenting practices (*PS*)
Khanna et al. (2017)	USA	Waitlist	Child Anxiety Tales	Website	Selective	49	NR	7 − 17 yrs	Child emotional problems (*CBCL, SCAS*)
Morgan et al. (2017)	Australia	Waitlist	Cool Little Kids Online	Website	Indicated	433	52.7	3 − 6 yrs	Child emotional problems (*PAS-R, SDQ*); Parents’ ineffective parenting practices (*OI/P*)
Palmer et al. (2023)	UK	Waitlist	Parent Positive App	App	Universal	646	48.5	4 − 10 yrs	Child behavior problems (*SDQ*); Child emotional problems (*SDQ*); Parents’ mental health problems (*DASS*)
Potharst et al. (2019)	Netherlands	Waitlist	Online Mindful Parenting Training	Website	Universal	76	56.0	3.5 yrs	Child behavior problems (*CBCL*); Child emotional problems (*CBCL*); Parents’ ineffective parenting practices (*PS*, *PSQ*); Parents’ mental health problems (*PHQ-4*)
Sanders et al. (2014)	New Zealand	No or minimal intervention	Triple P Online	Website	Indicated	193	33.0	3 − 8 yrs	Child behavior problems (*ECBI*); Parents’ ineffective parenting practices (*PS*); Parents’ mental health problems (*DASS*)
Sanders et al. (2012)	New Zealand	No or minimal intervention	Triple P Online	Website	Indicated	116	32.7	2 − 9 yrs	Child behavior problems (*ECBI*, *SDQ*); Child emotional problems (*SDQ*); Parents’ ineffective parenting practices (*PS*); Parents’ mental health problems (*DASS*)
Sim et al. (2020)	Australia	No or minimal intervention	Parenting Resilient Kids	Website	Universal	355	48.8	8 − 11 yrs	Child emotional problems (*RCADS-25*); Parents’ ineffective parenting practices (*PaRCADS*)
Sourander et al. (2018) Sourander et al. (2016)	Finland	No or minimal intervention	Strongest Families Smart Website	Website and weekly phone call	Indicated	464	38.1	4 yrs	Child behavior problems (*CBCL*); Child emotional problems (*CBCL*); Parents’ ineffective parenting practices (*PS*); Parents’ mental health problems (*DASS*)
Sung et al. (2021)	USA	No or minimal intervention	EMPOWER	Website	Indicated	301	NR	4 − 11 yrs	Parents’ ineffective parenting practices (*FASA*)
Tomlinson et al. (2023)	USA	Waitlist	Children in Between Two Families Now	Website	Selective	221	48.8	7.45 yrs	Parents’ mental health problems (*GMPS*)
Tuntipuchitanon et al. (2022)	Thailand	No or minimal intervention	Online Positive Parenting Programme	Videoconference	Universal	103	51.0	3 − 6 yrs	Child behavior problems (*SDQ*); Child emotional problems (*SDQ*); Parents’ ineffective parenting practices (*PSDQ*); Parents’ mental health problems (*PSS, PSI*)

*CAPES* Child Adjustment and Parent Efficacy Sale, *CBCL* Child Behavior Checklist, *CRPRQ* Child-Rearing Practice Report Questionnaire, *DASS* Depression, Anxiety and Stress Scale, *CERS* Conners Early Childhood Rating Scale, *ECBI* Eyberg Child Behavior Inventory, *EMA-EB* Ecological Momentary Assessment of Daily Externalizing Behavior, *FASA* Family Accommodation Scale – Anxiety, *GMPS* Global Measure of Perceived Stress, *OI/P* Over-Involved/Protective Parenting Scale, *PaRCADS* Parenting to Reduce Child Anxiety and Depression, *PARYC* Parenting Young Children, *PAS* Preschool Anxiety Scale, *PAS-R* Revised Preschool Anxiety Scale, *PHQ-4* Patient Health Questionnaire-4, *PPI* Parenting Practices Interview, *PQ* Parenting Questionnaire, *PS* Parenting Scale, *PSDQ* Parenting Styles and Dimensions Questionnaire, *PSI* Parenting Stress Index, *PSQ* Parental Stress Questionnaire, *PSS* Parental Stress Scale, *RCADS-25* Revised Children’s Anxiety and Depression Scale-25, *SCAS* Spence Children’s Anxiety Scale, *SCL-90* Symptom Checklist-90, *SDQ* Strengths and Difficulties Questionnaire, *SNAP-IV* Swanson, Nolan, and Pelham-IV, *NR* Not Reported, *USA* United States of America, *UK* United Kingdom

In terms of included outcomes, most trials estimated online parenting programs on child behavioral problems (18 trials on 13 different programs), and on child emotional problems (seven trials on seven programs). Parents’ parenting practices and mental health problems were examined less frequently (two trials on three programs for both outcomes). The programs to support parents with child behavior problems were mostly delivered through website (*n* = 9, 69.2%), self-directed (*n* = 7, 53.6%), and had 3 to 11 sessions or modules. The programs to support parents with child emotional problems were mostly delivered through website (*n* = 5, 71.4%), self-directed (*n* = 3, 42.9%), and had 1 to 12 sessions or modules. The programs to support parents with their parenting practices and mental health problems had 4 to 12 sessions or modules, were delivered through website and self-directed, with two being evaluated in the same trial (Tomlinson et al., 2023). Most trials compared the online parenting programs against a waitlist (*n* = 18, 66.7%) or a no or minimal intervention (*n* = 9, 33.3%) control condition.

The majority of the trials included parents of younger children aged 2 to 9, but some trials also included parents of early teens (DeGarmo & Jones, 2019; Enebrink et al., 2012; Khanna et al., 2017; Palmer et al., 2023; Sim et al., 2020; Sung et al., 2021). Most trials on online parenting programs for children’s behavioral problems included more parents of boys, whereas most trials on online parenting programs for children’s emotional problems included more parents of girls. Most of the trials were preregistered (*n* = 18, 66.67%) and did not report on whether included blinded outcome assessors since parents completed the evaluations autonomously online (*n* = 16, 59.3%). Less than half (*n* = 11, 40.74%) included additional follow-up assessments, ranging from 6 weeks (Tuntipuchitanon et al., 2022) to 2 years (Sourander et al., 2018) postintervention.

#### Online Parenting Programs’ Components

Online parenting programs’ characteristics including their clusters of components based on theoretical approaches are presented in Table 2. All the components identified in each program are presented in Table S2 (see Online Resource 5). Most programs targeting children’s behavior problems included clusters of components under four or more theoretical approaches (*n* = 18, 66.7%). The most common theoretical approaches were learning theory perspectives (*n* = 12, 92.3%), relationship perspectives (*n* = 11, 84.6%), parental self-care (*n* = 10, 76.9%), and psychoeducation (*n* = 9, 69.2%). Similarly, most programs targeting children’s emotional problems included clusters of components under four or more theoretical approaches (*n* = 5, 71.4%). All include parents as therapist and psychoeducation, and most of them also include components on parental self-care (*n* = 5, 71.4%) and relationship perspectives (*n* = 4, 36.4%). Programs targeting parenting practices and parent mental health often included fewer components. All include parental self-care.

**Table 2:** Online parenting programs, their main characteristics, and clusters of components under theoretical approaches

Online program	Focus of the program	Delivery method	Facilitator role	Number of sessions or modules	Theoretical approaches of the components in the program					
					PE	RP	LTp	PP	PSC	PT
Adventures in Parenting	Parents’ parenting practices and mental health problems	Website	None	12	-	✓	✓	✓	✓	✓
Child Anxiety Tales	Emotional problems	Website	None	10	✓	-	✓	-	✓	✓
Children in Between	Parents’ parenting practices and mental health problems	Website	None	6	✓	-	-	✓	✓	-
Confident Parent Internet Guide program	Behavior problems	Website	None	10	✓	-	✓	-	-	-
Cool Little Kids Online	Emotional problems	Website	Little	8	✓	✓	✓	-	✓	✓
EMPOWER	Emotional problems	Website	None	1	✓	-	-	-	-	✓
ezParent Program	Behavior problems	App	None	6	-	✓	✓	-	✓	-
Fathering Through Change	Behavior problems	Website	None	10	✓	✓	✓	✓	✓	✓
Group Executive Functioning and Online Parent Training	Behavior problems	Videoconference platform	Full	8	✓	✓	✓	✓	✓	-
iCALM Telehealth Program	Emotional problems	Videoconference platform	Full	12	✓	✓	✓	✓	✓	✓
Internet-based Parent Management Treatment	Behavior problems	Website	Little	7	-	✓	✓	-	-	-
Modified version of the BRAVE-ONLINE	Emotional problems	Website	Little	6	✓	-	-	-	✓	✓
Online Behavioral Parent Training	Behavior problems	Website	Little	10	-	✓	✓	-	✓	-
Online Mindful Parenting Training	Behavior problems	Website	None	8	✓	✓	-	-	✓	-
Online Positive Parenting Programme	Behavior problems	Videoconference platform	Full	8	✓	✓	✓	✓	✓	-
Parent Positive App	Behavior problems	App	Little	3	✓	✓	✓	-	✓	✓
Parenting Resilient Kids	Emotional problems	Website	None	12	✓	✓	-	✓	✓	✓
Self-Help Parenting Program	Behavior problems	Website	None	11	✓	✓	✓	✓	-	✓
Strongest Families Smart Website	Behavior problems	Website	Little	11	-	-	✓	-	✓	✓
Triple P Online	Behavior problems	Website	None	8	✓	✓	✓	✓	✓	✓
Triple P Online Brief	Behavior problems	Website	None	5	✓	✓	✓	✓	✓	✓
Turtle Program	Emotional problems	Videoconference platform	Full	8	✓	✓	-	-	-	✓
Two Families Now	Parents’ parenting practices and mental health problems	Website	None	4	-	-	-	-	✓	-

The components identified in each program are presented in Online Resource 5

*None* self-directed program, *Little* self-directed program with occasional contact with a facilitator, *Full* program delivered by a facilitator through videoconference, *PE* psychoeducation, *RP* relationship perspectives, *LTp* learning theory perspectives, *PP* proactive parenting, *PSC* parental self-care, *PT* parents as therapist

### Main Effects of Online Parenting Programs

The online parenting programs had significant overall effects on each of the outcome variables (Table 3). Online parenting programs reduced children’s behavioral problems, *d* =  − 0.52, 95% CI [− 0.95, − 0.09], and emotional problems, *d* =  − 0.53, 95% CI [− 1.05, 0.00], by around half a standard deviation each. Online parenting programs reduced parents’ ineffective parenting practices, *d* =  − 0.68, 95% CI [− 1.15, − 0.21], and mental health problems, *d* =  − 0.53, 95% CI [− 0.98, − 0.01], by more than half a standard deviation and half a standard deviation, respectively. However, heterogeneity between effect sizes and trials was high, suggesting that the overall effect should be interpreted with caution.

**Table 3:** Online parenting programs effects (Cohen’s *d*) on reduced child behavioral and emotional problems, parents’ ineffective parenting practices, and mental health problems

	SMD Cohen’s *d*	95% CI lower bound	95% CI upper bound	*τ*^*2*^	*k*	*n*
Child behavior problems	− 0.52	− 0.95	− 0.09	1.00	17	82
Child emotional problems	− 0.53	− 1.05	0.00	1.20	15	47
Parents’ ineffective parenting practices	− 0.68	− 1.15	− 0.21	1.00	18	95
Parents’ mental health problems	− 0.53	− 0.98	− 0.01	1.01	16	65

*τ*^*2*^ between-study variance, *k* number of trials, *n* number of effect sizes

### Relative Effects of Different Clusters of Components on Children’s Behavioral and Emotional Problems

**Fig. 2:**
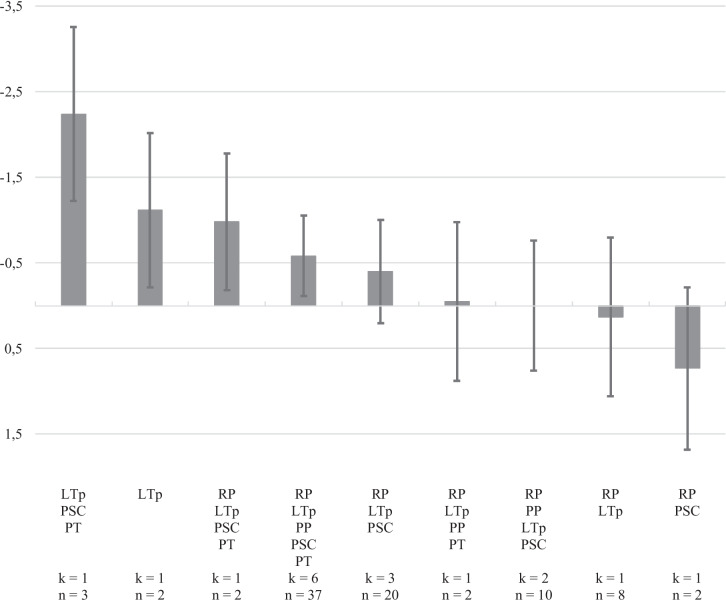
Relative effects (Cohen’s *d*) of different clusters of components compared with the control group on children’s behavioral problems

The network meta-analysis of programs for children’s behavioral problems included nine clusters of five components based on 17 studies (*τ*^2^ = 0.40; Fig. 2). The network of eligible comparisons of clusters of components (Figure S1, see Online Resource 6) suggests that programs with fewer components and those adopting a learning theory perspective, either in combination with parental self-care and parents as therapist or without any additional approaches, are most likely to yield the strongest effects. The overall effect size of these clusters was *d* =  − 2.24, 95% CI [− 3.26, − 1.22], and *d* =  − 1.12, 95% CI [− 2.02, − 0.23], respectively. Other clusters yielding overall significant effects reducing child behavioral problems in comparison to no or minimal intervention are a combination of relationship perspectives, learning theory perspectives, parental self-care, and parents as therapist, *d* =  − 0.98, 95% CI [− 1.78, − 0.19], and of relationship perspectives, learning theory perspectives, proactive parenting, parental self-care, and parents as therapist, *d* =  − 0.58, 95% CI [− 1.05, 0.11]. Clusters not yielding significant effect are those that do not include any learning theory-based content, those combining relationship perspectives with parental self-care, *d* = 0.73, 95% CI [− 0.22, 1.67], and those combining learning theory and relationship perspectives, *d* = 0.13, 95% CI [− 0.80, 1.06], with parental self-care, *d* =  − 0.40, 95% CI [− 1.00, 0.21], with parental self-care and proactive parenting, *d* = 0.00, 95% CI [− 0.76, 0.77], and proactive parenting with parents as therapist, *d* =  − 0.05, 95% CI [− 0.98, 0.87]. However, results should be interpreted with caution as some of the clusters are informed by one single study.

The relative ranking of the different clusters of components was compared with no or minimal intervention using *p*-scores, which are presented in Table S3 (see Online Resource 7). Online parenting programs combining components fitting learning theory perspectives, parental self-care, and parents as therapist approaches seem to be more likely to be successful in reducing child behavioral problems. Then, online parenting programs adopting only learning theory perspectives or combining relationship perspectives, learning theory perspectives, parental self-care, and parents as therapist, with or without proactive parenting, have the largest chance of being most effective in reducing child behavioral problems in comparison to no or minimal intervention.

**Fig. 3:**
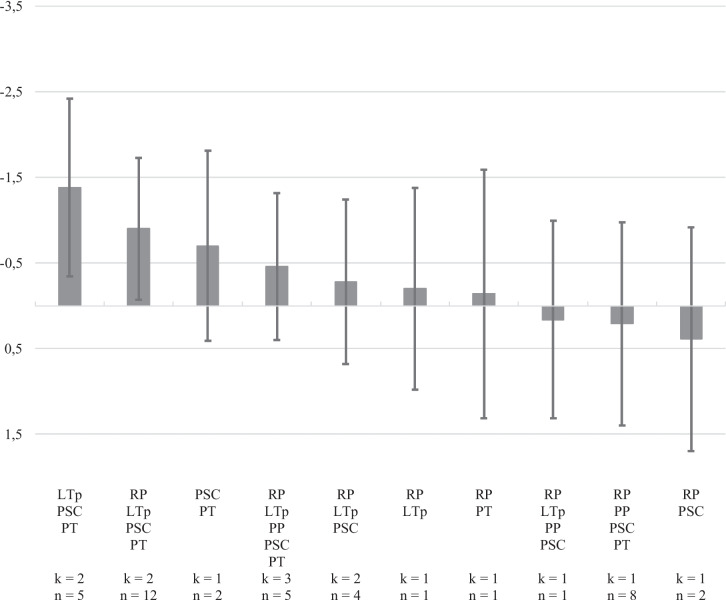
Relative effects (Cohen’s *d*) of different clusters of components compared with the control group on children’s emotional problems

The network meta-analysis of online parenting programs on child emotional problems included 10 clusters of five components based on 15 studies (*τ*^2^ = 0.81; Fig. 3). The network of eligible comparisons of clusters of components (Figure S2, see Online Resource 8) suggests that programs combining a learning theory perspective, parental self-care, and parents as therapist, with or without adding relationship perspectives to this, are most likely to yield the strongest effects. The overall effect size of these clusters was *d* =  − 1.38, 95%CI [− 2.42, − 0.33], without relationship perspectives, and *d* =  − 0.90, 95% CI [− 1.73, − 0.07], with relationship perspectives. Clusters not yielding significant effects are primarily those that do not include any learning theory-based content, and combine parental self-care and parents as therapist approaches, *d* =  − 0.70, 95% CI [− 1.81, 0.41], relationship perspectives with parents as therapist, *d* =  − 0.14, 95% CI [− 1.59, 1.31], or parental self-care, *d* = 0.39, 95% CI [− 0.92, 1.71], or that combine all four components, *d* = 0.21, 95% CI [− 0.98, 1.41]. And secondly, those combining components under learning theory and relationship perspectives, *d* =  − 0.20, 95% CI [− 1.38, 0.98], together with parental self-care, *d* =  − 0.28, 95% CI [− 1.24, 0.68], in addition to proactive parenting, *d* = 0.16, 95% CI [− 0.99, 1.31], and also parents as therapist, *d* =  − 0.46, 95% CI [− 1.32, 0.40]. Also here, results should be interpreted with caution as some clusters of components are informed by one single study.

The relative ranking of the different clusters of components was compared with no or minimal intervention using *p*-scores, which are presented in Table S4 (see Online Resource 9). Online parenting programs with clusters of components fitting learning theory perspectives, parental self-care, and parents as therapist approaches and those combining the same components with a relationship perspectives approach seem to be the only programs successful in reducing child emotional problems in comparison to no or minimal intervention.

### Risk of Bias and Publication Bias Assessment

The summary of the authors’ risk of bias judgments for each risk of bias domain for each trial and overall, across the risk of bias domains, is presented in Figure S3 and Figure S4 (see Online Resource 10). Most studies (*n* = 15, 53.6%) were rated as having some concerns or high risk of bias in three or more domains. For the domain of the randomization process, 19 studies were rated as having low risk and nine as having some concerns regarding allocation concealment or randomization procedures. For the domain regarding deviations from intended interventions, 17 studies were rated as having some concerns given that participants and the people delivering the program were aware of intervention groups, or some participants did not adhere to the assigned intervention regimen, even though the trial used an appropriate analysis to estimate the effect of adhering to intervention. For the domain on missing outcome data, 16 studies were rated as having low risk as these presented complete data or used statistical methods to address missing data. For the domain regarding the measurement of the outcome, 18 studies were coded as presenting some concerns due to lack of information on or lack of blindness of the evaluators, or lack of information on whether the measurement or ascertainment of the outcome could have differed between intervention groups. Finally, 19 studies were rated as having a low risk of bias for the selective reporting domain as all presented preregistered analytic plans fitting with the analysis described in the studies.

In terms of publication bias, the funnel plots in Figure S5, Figure S6, Figure S7, and Figure S8 (see Online Resource 11) show that the distribution of the effect sizes is fairly symmetrical. Most effect sizes fall in the funnel, but there are outliers with particularly strong effects favoring the intervention: Baker et al. (2017), Sim et al. (2020), and Sourander et al. (2016, 2018). This suggests that some of our effect estimates might be inflated. However, effect size variance did not predict effect size magnitude, suggesting no evidence for significant publication bias.

## Discussion

Our meta-analysis sought to deepen the understanding of what content of parenting programs is more suitable to be delivered online. It did so by estimating the effects of online parenting programs on child behavioral and emotional problems, parents’ ineffective parenting practices and mental health problems, and by identifying how different clusters of components affect child behavioral and emotional problems.

The online parenting programs had moderate effects on reduced child behavioral and emotional problems and parents’ ineffective parenting practices and mental health problems. These effect sizes, which were reasonably similar across outcomes, are in the same direction and slightly higher than those from previous meta-analyses of the effects of online parenting programs (David et al., 2023; Florean et al., 2020; Opie et al., 2024; Spencer et al., 2020; Thongseiratch et al., 2020), and of meta-analyses addressing the effects of parenting programs delivered in person (e.g., Schwartz et al., 2019).

Our results suggest that online parenting programs are promising tools for supporting parents dealing with child behavioral and emotional problems, ineffective parenting practices, or mental health problems. However, heterogeneity was large, and our results may also be somewhat influenced by some of the characteristics of the included studies. Only one study included participants with socioeconomic disadvantages (Breitenstein et al., 2016), which may suggest that the overall participants had more resources and were perhaps exposed to fewer stressors. Also, dropout rates ranged from 0% (Donovan & March, 2014) to 61% (Sung et al., 2021), with 11 studies reporting dropout rates of more than 20%. Although most studies analyzed their data intention to treat, the reported mean scores may rely primarily on data from parents who completed the programs.

That heterogeneity in effect sizes within and between trials was high suggests that the overall effects should be interpreted with caution. Indeed, the evaluated programs vary profoundly in their contents and how they are delivered, as are the participants receiving them (e.g., some programs have multiple contents delivered by a facilitator in several sessions through videoconference platforms to parents of children with behavioral or emotional disorders, whereas other programs have fewer contents and are self-directed, available in websites to all parents).

We attempted to understand the heterogeneity in our network meta-analyses. Our findings suggest that online parenting programs with fewer components and those adopting a learning theory perspective, either in combination with parental self-care and parents as therapist or without additional approaches, are most likely to yield the strongest effects on child behavioral problems. These results are in line with prior findings that adopting a learning theory approach (e.g., contingent reinforcement of desirable or undesirable behavior; McAloon & de la Poer Beresford, 2023) and parental self-care (e.g., parental self-emotion regulation) is more effective in reducing child behavioral problems than for example teaching parents how to support the child or having additional phone calls to the program (Thongseiratch et al., 2020). The changes in children’s behavioral problems may take place through parents’ experiential learning, which gives them a set of skills that help them promote children’s adequate behavior and manage children’s misbehavior. This ultimately supports parents in regulating their own behavior, reducing parenting stress, and improving their family well-being.

Our findings have also shown that programs including a learning theory approach, parental self-care, and parents as therapist components, combined or in addition to relationship perspectives (e.g., contents for both the parent and child, focusing on their relationship; Costantini et al., 2023), also seem to be more effective in reducing child emotional problems. Parents’ experiential learning paired with a focus on their well-being, specific strategies to teach their child skills that will enhance their mental health and strengthen the parent–child relationship, contribute to support children in reducing their emotional problems.

The relative effect of the online parenting programs including relationship perspectives and proactive parenting components is smaller, unlike the findings reported in some network meta-analyses addressing in-person parenting programs’ components (Kjøbli et al., 2023; Tehrani et al., 2023). These contents, which include the promotion of positive involvement, child-led activities, mind-mindedness or empathy, direct commands, and clear limits, may be more difficult to implement in online programs, particularly in self-directed programs without facilitator contact, as were most of the programs included in our review.

Beyond the high heterogeneity, we acknowledge the limited number of studies per cluster of components. Our work is a first attempt at cataloging the theoretical approaches that online parenting programs are based on and exploring if there are any patterns where the programs’ effects on child behavioral and emotional problems vary by these combinations of theoretical approaches. The limited number of studies per cluster of components may be too small to draw conclusions about individual combinations of components. In addition, as the data extracted from the studies was based on the comparison between online programs and passive control conditions, the comparison of effects of the different clusters reported is an indirect one.

In terms of implications for practice, our work suggests online parenting programs may be an effective tool for professionals and parents to improve children’s behavioral and emotional problems, parents’ ineffective parenting practices, and mental health. Acknowledging that we had a limited number of studies for some of the clusters of components identified, our results provide preliminary evidence that children with behavioral problems and children with emotional problems benefit from slightly different program content. There seemed to be a robust trend that including learning theory-based components (i.e., with positive reinforcement contents, where parents learn to react to positive child behavior with praise and/or rewards, and non-violent disciplining techniques contents, where parents learn to react to disruptive child behavior with a nonviolent consequence to reduce the behavior) predicted the strongest effects in child behavioral problems. For child emotional problems, results suggest that a combination of learning theory perspectives, parental self-care (e.g., parental stress reduction, emotion regulation, and problem-solving skills), and parents as therapist (e.g., where parents teach their children emotion regulation and problem-solving skills and help them manage avoidance behaviors) predict the strongest effects. These findings can inform online parenting program developers on the contents to include targeting child behavioral and emotional problems.

Future research should identify the circumstances that allow parents and children to benefit more from online parenting programs, or from specific content in these programs. Individual participant data meta-analysis can help identify whether the effects of the programs and their components vary according to parents’ or children’s individual characteristics. Additional component network meta-analyses are needed to conduct more direct comparisons of the effects of different clusters of components within trials, and also compare the effects of the components of parenting programs delivered online and in person. Despite the evidence showing that online parenting programs are promising tools to support parents at substantially fewer costs (Florean et al., 2020; Ingels et al., 2022), these may not be suitable for all families. In fact, while some studies suggest that parents, on average, prefer online delivery over in-person delivery (Leijten et al., 2024), other studies find the opposite to be true (Engelbrektsson et al., 2023). Future research should address the individual differences between families to determine under which circumstances online programs can be used, and when do parents benefit more from in-person programs.

## Supplementary Information

Below is the link to the electronic supplementary material.

Supplementary file1 (DOCX 1.14 MB)

## Data Availability

The code used to perform the network meta-analyses is provided in supplemental material (see Online Resource 4).

## References

[CR1] Baker, S., Sanders, M. R., Turner, K. M. T., & Morawska, A. (2017). A randomized controlled trial evaluating a low-intensity interactive online parenting intervention, Triple P Online Brief, with parents of children with early onset conduct problems. *Behaviour Research and Therapy,**91*, 78–90. 10.1016/j.brat.2017.01.016 28167330 10.1016/j.brat.2017.01.016

[CR2] Backhaus, S., Leijten, P., Jochim, J., Melendez-Torres, G. J., & Gardner, F. (2023). Effects over time of parenting interventions to reduce physical and emotional violence against children: A systematic review and meta-analysis. *EClinicalMedicine,**60*, 102003. 10.1016/j.eclinm.2023.102003 37251634 10.1016/j.eclinm.2023.102003 PMC10209692

[CR3] Breitenstein, S. M., Fogg, L., Ocampo, E. V., Acosta, D. I., & Gross, D. (2016). Parent use and efficacy of a self-administered, tablet-based parent training intervention: A randomized controlled trial. *JMIR mHealth and uHealth,**4*(2), e36. 10.2196/mhealth.5202 27098111 10.2196/mhealth.5202 PMC4867750

[CR4] Breitenstein, S. M., Fehrenbacher, C., Holod, A. F., & Schoeny, M. E. (2021). A randomized trial of digitally delivered, self-administered parent training in primary care: Effects on parenting and child behavior. *The Journal of Pediatrics,**231*, 207–214. 10.1016/j.jpeds.2020.12.016 33338496 10.1016/j.jpeds.2020.12.016 PMC9272894

[CR5] Canário, A., Byrne, S., Creasey, N., Kodysova, E., Akik, B., Lewandowska-Walter, A., Stanke, K., Pecnik, N., & Leijten, P. (2022). The use of information and communication technologies in family support across Europe: A narrative review. *International Journal of Environmental Research and Public Health,**19*(3), 1488. 10.3390/ijerph19031488 35162511 10.3390/ijerph19031488 PMC8834894

[CR6] Canário, C., Abreu, L. I., Santos, S., Silva, M. M., Campos, J., Enes Rodrigues, C., Tavares, M., Mansilha, H., Torres, S., Serra Lemos, M., & Cruz, O. (2021). Delivering Group Lifestyle Triple P through digital practice: A case study with Portuguese parents. *Journal of Family Therapy,**43*(2), 232–255. 10.1111/1467-6427.12334

[CR7] Caspi, A., & Moffitt, T. E. (2018). All for one and one for all: Mental disorders in one dimension. *The American Journal of Psychiatry,**175*(9), 831–844. 10.1176/appi.ajp.2018.17121383 29621902 10.1176/appi.ajp.2018.17121383 PMC6120790

[CR8] Chu, L., Zhu, P., Ma, C., Pan, L., Shen, L., Wu, D., Wang, Y., & Yu, G. (2022). Effects of combing group executive functioning and online parent training on school-aged children with ADHD: A randomized controlled trial. *Frontiers in Pediatrics,**9*, 813305. 10.3389/fped.2021.813305 35223713 10.3389/fped.2021.813305 PMC8874140

[CR9] Comer, J. S., Furr, J. M., del Busto, C., Silva, K., Hong, N., Poznanski, B., Sanchez, A., Cornacchio, D., Herrera, A., Coxe, S., Miguel, E., Georgiadis, C., Conroy, K., & Puliafico, A. (2021). Therapist-led, internet-delivered treatment for early child social anxiety: A waitlist-controlled evaluation of the iCALM Telehealth program. *Behavior Therapy,**52*(5), 1171–1187. 10.1016/j.beth.2021.01.004 34452671 10.1016/j.beth.2021.01.004

[CR10] Costantini, I., López-López, J. A., Caldwell, D., Campbell, A., Hadjipanayi, V., Cantrell, S. J., Thomas, T., Badmann, N., Paul, E., James, D. M., Cordero, M., Jewell, T., Evans, J., & Pearson, R. M. (2023). Early parenting interventions to prevent internalising problems in children and adolescents: A global systematic review and network meta-analysis. *BMJ Mental Health,**26*(1), e300811. 10.1136/bmjment-2023-300811 37907332 10.1136/bmjment-2023-300811 PMC10619111

[CR11] Day, J. J., & Sanders, M. R. (2018). Do parents benefit from help when completing a self-guided parenting program online? A randomized controlled trial comparing Triple P Online with and without telephone support. *Behavior Therapy,**49*(6), 1020–1038. 10.1016/j.beth.2018.03.002 30316482 10.1016/j.beth.2018.03.002

[CR12] David, O. A., Fodor, L. A., Dascăl, M. D., & Miron, I. S. (2023). The efficacy of online parenting interventions in addressing emotional problems in children and adolescents: A meta-analysis of randomized controlled trials. *International Journal of Social Psychiatry,**69*(5), 1100–1112. 10.1177/00207640231156034 36860086 10.1177/00207640231156034

[CR13] David, O. A., Iuga, I. A., & Miron, I. S. (2024). Parenting: There is an app for that. A systematic review of parenting interventions apps. *Children and Youth Services Review,**156*, 107385. 10.1016/j.childyouth.2023.107385

[CR14] DeGarmo, D. S., & Jones, J. A. (2019). Fathering Through Change (FTC) intervention for single fathers: Preventing coercive parenting and child problem behaviors. *Development and Psychopathology,**31*(5), 1801–1811. 10.1017/S0954579419001019 31489831 10.1017/S0954579419001019

[CR15] de Jong, S., van den Hoofdakker, B., Van Der Veen-Mulders, L., Veenman, B., Twisk, J., Oosterlaan, J., & Luman, M. (2023). The efficacy of a self-help parenting program for parents of children with externalizing behavior: A randomized controlled trial. *European Child & Adolescent Psychiatry,**32*(10), 2031–2042. 10.1007/s00787-022-02028-0 35794395 10.1007/s00787-022-02028-0 PMC9261243

[CR16] Donovan, C., & March, S. (2014). Online CBT for preschool anxiety disorders: A randomised control trial. *Behaviour Research and Therapy,**58*, 24–35. 10.1016/j.brat.2014.05.001 24927471 10.1016/j.brat.2014.05.001

[CR17] DuPaul, G. J., Kern, L., Belk, G., Custer, B., Daffner, M., Hatfield, A., & Peek, D. (2018). Face-to-face versus online behavioral parent training for young children at risk for ADHD: Treatment engagement and outcomes. *Journal of Clinical Child and Adolescent Psychology,**47*(S1), S369–S383. 10.1080/15374416.2017.1342544 28715272 10.1080/15374416.2017.1342544

[CR18] Ehrensaft, M. K., Knous-Westfall, H. M., & Alonso, T. L. (2016). Web-based prevention of parenting difficulties in young, urban mothers enrolled in post-secondary education. *The Journal of Primary Prevention,**37*(6), 527–542. 10.1007/s10935-016-0448-1 27624608 10.1007/s10935-016-0448-1

[CR19] Enebrink, P., Högström, J., Forster, M., & Ghaderi, A. (2012). Internet-based parent management training: A randomized controlled study. *Behaviour Research and Therapy,**50*(4), 240–249. 10.1016/j.brat.2012.01.006 22398153 10.1016/j.brat.2012.01.006

[CR20] Engelbrektsson, J., Salomonsson, S., Högström, J., Sorjonen, K., Sundell, K., & Forster, M. (2023). Parent training via internet or in group for disruptive behaviors: A randomized clinical noninferiority trial. *Journal of the American Academy of Child & Adolescent Psychiatry,**62*(9), 987–997. 10.1016/j.jaac.2023.01.019 36863414 10.1016/j.jaac.2023.01.019

[CR21] Farris, J. R., Bert, S. S. C., Nicholson, J. S., Glass, K., & Borkowski, J. G. (2013). Effective intervention programming: Improving maternal adjustment through parent education. *Administration and Policy in Mental Health,**40*(3), 211–223. 10.1007/s10488-011-0397-1 22246615 10.1007/s10488-011-0397-1

[CR22] Florean, I. S., Dobrean, A., Pasarelu, C. R., Georgescu, R. D., & Milea, I. (2020). The efficacy of internet-based parenting programs for children and adolescents with behavior problems: A meta-analysis of randomized clinical trials. *Clinical Child and Family Psychology Review,**23*(4), 510–528. 10.1007/s10567-020-00326-0 32897527 10.1007/s10567-020-00326-0

[CR23] Franke, N., Keown, L. J., & Sanders, M. R. (2020). An RCT of an online parenting program for parents of preschool-aged children with ADHD symptoms. *Journal of Attention Disorders,**24*(12), 1716–1726. 10.1177/1087054716667598 27609783 10.1177/1087054716667598

[CR24] Guedes, M., Maia, R., Matos, I., Antunes, M., Rolão, T., Chronis-Tuscano, A., Rubin, K. H., Veríssimo, M., & Santos, A. J. (2023). Preliminary perceived intervention changes and engagement in an evidence-based program targeted at behavioral inhibition during early childhood, delivered in-person and online. *Frontiers in Psychology,**14*, 1187255. 10.3389/fpsyg.2023.1187255 37303908 10.3389/fpsyg.2023.1187255 PMC10254805

[CR25] Hedges, L. V., Tipton, E., & Johnson, M. C. (2010). Robust variance estimation in meta-regression with dependent effect size estimates. *Research Synthesis Methods,**1*(1), 39–65. 10.1002/jrsm.5 26056092 10.1002/jrsm.5

[CR26] Hutchings, J., Owen, D. A., & Williams, M. E. (2023). Development and initial evaluation of the COnfident Parent INternet Guide program for parents of 3–8 year olds. *Frontiers in Psychology,**14*, 1228144. 10.3389/fpsyg.2023.1228144 37560109 10.3389/fpsyg.2023.1228144 PMC10408448

[CR27] Hutton, B., Salanti, G., Caldwell, D. M., Chaimani, A., Schmid, C. H., Cameron, C., Ioannidis, J. P. A., Straus, S., Thorlund, K., Jansen, J. P., Mulrow, C., Catalá-López, F., Gøtzsche, P. C., Dickersin, K., Boutron, I., Altman, D. G., & Moher, D. (2015). The PRISMA extension statement for reporting of systematic reviews incorporating network meta-analyses of health care interventions: Checklist and explanations. *Annals of Internal Medicine,**162*(11), 777–784. 10.7326/M14-2385 26030634 10.7326/M14-2385

[CR28] Ingels, J. B., Corso, P. S., Prinz, R. J., Metzler, C. W., & Sanders, M. R. (2022). Online-delivered over staff-delivered parenting intervention for young children with disruptive behavior problems: Cost-minimization analysis. *JMIR Pediatrics and Parenting,**5*(1), e30795. 10.2196/30795 35275084 10.2196/30795 PMC8956984

[CR29] Jervis, P., Coore-Hall, J., Pitchik, H. O., Arnold, C. D., Grantham-McGregor, S., Rubio-Codina, M., Baker-Henningham, H., Fernald, L. C. H., Hamadani, J., Smith, J. A., Trias, J., & Walker, S. P. (2023). The reach up parenting program, child development, and maternal depression: A meta-analysis. *Pediatrics,**151*(S2), e2023060221D. 10.1542/peds.2023-060221D 37125892 10.1542/peds.2023-060221D

[CR30] Jolstedt, M., Wahlund, T., Lenhard, F., Ljotsson, B., Mataix-Cols, D., Nord, M., Ost, L.-G., Hogstrom, J., Serlachius, E., & Vigerland, S. (2018). Efficacy and cost-effectiveness of therapist-guided internet cognitive behavioural therapy for paediatric anxiety disorders: A single-centre, single-blind, randomised controlled trial. *The Lancet Child & Adolescent Health,**2*(11), 792–801. 10.1016/S2352-4642(18)30275-X 30241993 10.1016/S2352-4642(18)30275-X

[CR31] Jones, S. H., Jovanoska, J., Calam, R., Wainwright, L. D., Vincent, H., Asar, O., Diggle, P. J., Parker, R., Long, R., Sanders, M., & Lobban, F. (2017). Web-based integrated bipolar parenting intervention for parents with bipolar disorder: A randomised controlled pilot trial. *Journal of Child Psychology and Psychiatry,**58*(9), 1033–1041. 10.1111/jcpp.12745J 28512921 10.1111/jcpp.12745 PMC5573909

[CR32] Kaminski, J., Valle, L. A., Filene, J. H., & Boyle, C. L. (2008). A meta-analytic review of components associated with parent training program effectiveness. *Journal of Abnormal Child Psychology,**36*(4), 567–589. 10.1007/s10802-007-9201-9 18205039 10.1007/s10802-007-9201-9

[CR33] Khanna, M. S., Carper, M. M., Harris, M. S., & Kendall, P. C. (2017). Web-based parent-training for parents of youth with impairment from anxiety. *Evidence-Based Practice in Child and Adolescent Mental Health,**2*(1), 43–53. 10.1080/23794925.2017.1283548 29270464 10.1080/23794925.2017.1283548 PMC5734653

[CR34] Kjøbli, J., Melendez, T. G. J., Gardner, F., Backhaus, S., Linnerud, S., & Leijten, P. (2023). Research review: Effects of parenting programs for children’s conduct problems on children’s emotional problems – a network meta-analysis. *Journal of Child Psychology & Psychiatry,**64*(3), 348–356. 10.1111/jcpp.13697 36097742 10.1111/jcpp.13697 PMC10087885

[CR35] Leclair Mallette, I., Letarte, M., Hélie, S., Sicotte, R., & Temcheff, C. E. (2021). Is the Incredible Years parenting programme predictive of case closure in child protection services for neglect? A quasi-experimental study. *Child & Family Social Work,**26*(4), 687–695. 10.1111/cfs.12849

[CR36] Leijten, P., Melendez, T. G. J., & Gardner, F. (2022). Research review: The most effective parenting program content for disruptive child behavior – a network meta-analysis. *Journal of Child Psychology & Psychiatry,**63*(2), 132–142. 10.1111/jcpp.13483 34240409 10.1111/jcpp.13483

[CR37] Leijten, P., Rienks, K., Groenman, A. P., Anand, M., KömürcüAkik, B., David, O., Kızıltepe, R., Thongseiratch, T., & Canário, A. C. (2024). Online parenting support: Meta-analyses of non-inferiority and additional value to in-person support. *Children and Youth Services Review,**159*, 107497. 10.1016/j.childyouth.2024.107497

[CR38] McAloon, J., & de la Poer Beresford, K. (2023). Online behavioral parenting interventions for disruptive behavioral disorders: A PRISMA based systematic review of clinical trials. *Child Psychiatry & Human Development,**54*(2), 379–396. 10.1007/s10578-021-01253-z 34561755 10.1007/s10578-021-01253-z

[CR39] McGuinness, L. A., & Higgins, J. P. T. (2021). Risk-of-bias VISualization (robvis): An R package and shiny web app for visualizing risk-of-bias assessments. *Research Synthesis Methods,**12*(1), 55–61. 10.1002/jrsm.1411 32336025 10.1002/jrsm.1411

[CR40] Melendez-Torres, G. J., Bonell, C., & Thomas, J. (2015). Emergent approaches to the meta-analysis of multiple heterogeneous complex interventions. *BMC Medical Research Methodology,**15*, 47. 10.1186/s12874-015-0040-z 26032785 10.1186/s12874-015-0040-z PMC4455278

[CR41] Moher, D., Liberati, A., Tetzlaff, J., & Altman, D. G. (2009). Preferred reporting items for systematic reviews and meta-analyses: The PRISMA statement. *British Medical Journal,**339*(7716), 332–336. 10.1136/bmj.b2535 10.1136/bmj.b2535 PMC2714657 19622551

[CR42] Morgan, A. J., Rapee, R. M., Salim, A., Goharpey, N., Tamir, E., McLellan, L. F., & Bayer, J. K. (2017). Internet-delivered parenting program for prevention and early intervention of anxiety problems in young children: Randomized controlled trial. *Journal of the American Academy of Child and Adolescent Psychiatry,**56*(5), 417–425. 10.1016/j.jaac.2017.02.010 28433091 10.1016/j.jaac.2017.02.010

[CR43] Opie, J. E., Esler, T. B., Clancy, E. M., Wright, B., Painter, F., Vuong, A., Booth, A. T., Newman, L., Johns-Hayden, A., Hameed, M., Hooker, L., Olsson, C., & McIntosh, J. E. (2024). Universal digital programs for promoting mental and relational health for parents of young children: A systematic review and meta-analysis. *Clinical Child and Family Psychology Review,**27*, 23–52. 10.1007/s10567-023-00457-0 37917315 10.1007/s10567-023-00457-0 PMC10920439

[CR44] Ouzzani, M., Hammady, H., Fedorowicz, Z., & Elmagarmid, A. (2016). Rayyan – a web and mobile app for systematic reviews. *Systematic Reviews,**5*, 1–10. 10.1186/s13643-016-0384-4 27919275 10.1186/s13643-016-0384-4 PMC5139140

[CR45] Page, M. J., McKenzie, J. E., Bossuyt, P. M., Boutron, I., Hoffmann, T. C., Mulrow, C. D., Shamseer, L., Tetzlaff, J. M., Akl, E. A., Brennan, S. E., Chou, R., Glanville, J., Grimshaw, J. M., Hróbjartsson, A., Lalu, M. M., Li, T., Loder, E. W., Mayo-Wilson, E., McDonald, S., … Moher, D. (2021). The PRISMA 2020 statement: an updated guideline for reporting systematic reviews. *Systematic Reviews*, *10*(1), 1–11. 10.1186/s13643-021-01626-4 10.1186/s13643-021-01626-4 PMC8008539 33781348

[CR46] Palmer, M., Beckley-Hoelscher, N., Shearer, J., Kostyrka-Allchorne, K., Robertson, O., Koch, M., Pearson, O., Slovak, P., Day, C., Byford, S., Goldsmith, K., Waite, P., Creswell, C., & Sonuga-Barke, E. J. S. (2023). The effectiveness and cost-effectiveness of a universal digital parenting intervention designed and implemented during the COVID-19 pandemic: Evidence from a rapid-implementation randomized controlled trial within a cohort. *Journal of Medical Internet Research,**25*, e44079. 10.2196/44079 37498669 10.2196/44079 PMC10415938

[CR47] Prinz, R. J. (2019). A population approach to parenting support and prevention: The Triple P system. *The Future of Children,**29*(1), 122–143. 10.1353/foc.2019.0005

[CR48] Potharst, E. S., Boekhorst, M. G. B. M., Cuijlits, I., van Broekhoven, K. E. M., Jacobs, A., Spek, V., Nyklicek, I., Bogels, S. M., & Pop, V. J. M. (2019). A randomized control trial evaluating an online mindful parenting training for mothers with elevated parental stress. *Frontiers in Psychology,**10*, 1550. 10.3389/fpsyg.2019.01550 31379646 10.3389/fpsyg.2019.01550 PMC6650592

[CR49] RStudio Team. (2020). *RStudio: Integrated development for R. RStudio*, Boston, MA: RStudio, PBC. http://www.rstudio.com/

[CR50] Sanders, M. R., Baker, S., & Turner, K. M. T. (2012). A randomized controlled trial evaluating the efficacy of Triple P Online with parents of children with early-onset conduct problems. *Behaviour Research and Therapy,**50*(11), 675–684. 10.1016/j.brat.2012.07.004 22982082 10.1016/j.brat.2012.07.004

[CR51] Sanders, M. R., Dittman, C. K., Farruggia, S. P., & Keown, L. J. (2014). A comparison of online versus workbook delivery of a self-help positive parenting program. *The Journal of Primary Prevention,**35*(3), 125–133. 10.1007/s10935-014-0339-2 24500106 10.1007/s10935-014-0339-2

[CR52] Schwartz, C., Barican, J. L., Yung, D., Zheng, Y., & Waddell, C. (2019). Six decades of preventing and treating childhood anxiety disorders: A systematic review and meta-analysis to inform policy and practice. *Evidence-Based Mental Health,**22*(3), 103–110. 10.1136/ebmental-2019-300096 31315926 10.1136/ebmental-2019-300096 PMC6663062

[CR53] Schwarzer, G., Carpenter, J. R., & Rücker, G. (2015). *Meta-analysis with R*. Springer International Publishing. https://researchonline.lshtm.ac.uk/id/eprint/2319347/. Accessed 5 Jan 2024

[CR54] Sim, W. H., Fernando, L. M. N., Jorm, A. F., Rapee, R. M., Lawrence, K. A., Mackinnon, A. J., & Yap, M. B. H. (2020). A tailored online intervention to improve parenting risk and protective factors for child anxiety and depression: Medium-term findings from a randomized controlled trial. *Journal of Affective Disorders,**277*, 814–824. 10.1016/j.jad.2020.09.019 33065822 10.1016/j.jad.2020.09.019

[CR55] Sourander, A., McGrath, P. J., Ristkari, T., Cunningham, C., Huttunen, J., Lingley-Pottie, P., Hinkka-Yli-Salomäki, S., Kinnunen, M., Vuorio, J., Sinokki, A., Fossum, S., & Unruh, A. (2016). Internet-assisted parent training intervention for disruptive behavior in 4-year-old children: A randomized clinical trial. *JAMA Psychiatry,**73*(4), 378–387. 10.1001/jamapsychiatry.2015.3411 26913614 10.1001/jamapsychiatry.2015.3411

[CR56] Sourander, A., McGrath, P. J., Ristkari, T., Cunningham, C., Huttunen, J., Hinkka-Yli-Salomäki, S., Kurki, M., & Lingley-Pottie, P. (2018). Two-year follow-up of internet and telephone assisted parent training for disruptive behavior at age 4. *Journal of the American Academy of Child & Adolescent Psychiatry,**57*(9), 658–668. 10.1016/j.jaac.2018.07.001 30196869 10.1016/j.jaac.2018.07.001

[CR57] Spencer, C. M., Topham, G. L., & King, E. L. (2020). Do online parenting programs create change?: A meta-analysis. *Journal of Family Psychology,**34*(3), 364–374. 10.1037/fam0000605 31697102 10.1037/fam0000605

[CR58] StataCorp. (2019). *Stata Statistical software: Release 16*. StataCorp LLC.

[CR59] Sterne, J. A. C., Savović, J., Page, M. J., Elbers, R. G., Blencowe, N. S., Boutron, I., Cates, C. J., Cheng, H. Y., Corbett, M. S., Eldridge, S. M., Emberson, J. R., Hernán, M. A., Hopewell, S., Hróbjartsson, A., Junqueira, D. R., Jüni, P., Kirkham, J. J., Lasserson, T., Li, T., … Higgins, J. P. T. (2019). RoB 2: a revised tool for assessing risk of bias in randomised trials. *British Medical Journal*, *366*, l4898. 10.1136/bmj.l4898 10.1136/bmj.l4898 31462531

[CR60] Sullivan, A. D. W., Forehand, R., Acosta, J., Parent, J., Comer, J. S., Loiselle, R., & Jones, D. J. (2021). COVID-19 and the acceleration of behavioral parent training telehealth: Current status and future directions. *Cognitive and Behavioral Practice,**28*(4), 618–629. 10.1016/j.cbpra.2021.06.012 34629838 10.1016/j.cbpra.2021.06.012 PMC8488182

[CR61] Sung, J. Y., Mumper, E., & Schleider, J. L. (2021). Empowering anxious parents to manage child avoidance behaviors: Randomized control trial of a single-session intervention for parental accommodation. *JMIR Mental Health,**8*(7). 10.2196/29538 10.2196/29538 PMC8292931 34255718

[CR62] Tehrani, H. D., Yamini, S., & Vazsonyi, A. T. (2023). The effectiveness of parenting program components on disruptive and delinquent behaviors during early and middle childhood: A component network meta-analysis. *Journal of Experimental Criminology*. 10.1007/s11292-023-09562-0

[CR63] Thomas, R., & Zimmer-Gembeck, M. J. (2007). Behavioral outcomes of parent–child interaction therapy and Triple P—positive parenting program: A review and meta-analysis. *Journal of Abnormal Child Psychology,**35*(3), 475–495. 10.1007/s10802-007-9104-9 17333363 10.1007/s10802-007-9104-9

[CR64] Thongseiratch, T., Leijten, P., & Melendez-Torres, G. J. (2020). Online parent programs for children’s behavioral problems: A meta-analytic review. *European Child & Adolescent Psychiatry,**29*(11), 1555–1568. 10.1007/s00787-020-01472-0 31925545 10.1007/s00787-020-01472-0

[CR65] Tomlinson, C. S., Rudd, B. N., Applegate, A. G., Diaz, A., & Holtzworth-Munroe, A. (2023). Evaluation of court-initiated randomized controlled trial of online parent programs for divorcing and separating parents. *Journal of Family Psychology,**37*(1), 65–78. 10.1037/fam0001049 36441999 10.1037/fam0001049

[CR66] Tuntipuchitanon, S., Kangwanthiti, I.-O., Jirakran, K., Trairatvorakul, P., & Chonchaiya, W. (2022). Online positive parenting programme for promoting parenting competencies and skills: Randomised controlled trial. *Scientific Reports,**12*, 6420. 10.1038/s41598-022-10193-0 35440798 10.1038/s41598-022-10193-0 PMC9017087

[CR67] Viechtbauer, W. (2010). Conducting meta-analyses in R with the metafor package. *Journal of Statistical Software,**36*(3), 1–48. 10.18637/jss.v036.i03

