# Supplementary material for: Online Parenting Programs for Children’s Behavioral and Emotional Problems: a Network Meta-Analysis
Source: Prev Sci. 2024 Oct 13;26(4):592–609. doi: 10.1007/s11121-024-01735-1 (PMC12209006; doi:10.1007/s11121-024-01735-1)
Supplement: Supplementary file 1 — Supplementary file1 (DOCX 1.14 MB) [file 11121_2024_1735_MOESM1_ESM.docx]

**Supplementary Material**

**Online Parenting Programs for Children’s Behavioral and Emotional Problems: A Network Meta-Analysis** (https://doi.org/10.1007/s11121-024-01735-1)

Online Resources

| 1 | PRISMA 27-item Checklist……………………………………………………………………… | p. 2 |
| --- | --- | --- |
| 2 | Search String Used in Each Database…………………………………………………………… | p. 4 |
| 3 | Table S1. Online Parenting Programs’ Components: Theoretical Approaches and Contents….. | p. 6 |
| 4 | Scripts Used to Performed Network Meta-Analysis to Compare the Effects of Each Cluster of Components on Child Behavioral Problems……………………………………………………. | p. 8 |
|  | Scripts Used to Performed Network Meta-Analysis to Compare the Effects of Each Cluster of Components on Child Emotional Problems…………………………………………………….. | p. 9 |
| 5 | Table S2. Components and Respective Theoretical Approach Identified in Each Online Parenting Program……………………………………………………………………………….. | p. 10 |
| 6 | Figure S1. Network Plot of Eligible Comparisons of Clusters of Components for Child Behavioral Problems……………………………………………………………………………... | p. 12 |
| 7 | Table S3. Ranking of Clusters of Components from the Cluster Most Likely to be Most Effective to the Cluster Least Likely to be Most Effective in Reducing Children’s Behavioral Problems…………………………………………………………………………………………. | p. 13 |
| 8 | Figure S2. Network Plot of Eligible Comparisons of Clusters of Components for Child Emotional Problems……………………………………………………………………………... | p. 14 |
| 9 | Table S4. Ranking of Clusters of Components from the Cluster Most Likely to be Most Effective to the Cluster Least Likely to be Most Effective in Reducing Children’s Emotional Problems…………………………………………………………………………………………. | p.15 |
| 10 | Figure S3. Risk of Bias Traffic Light Plot at the Study Level Depicting Review Authors' Judgments about Each Risk of Bias……………………………………………………………... | p. 16 |
|  | Figure S4. Risk of Bias Summary Plot Depicting Review Authors' Judgments About Each Risk of Bias Item Presented as Percentages across all Included Studies……………………………… | p. 17 |
| 11 | Figure S5. Funnel Plot for Child Behavioral Problems………………………………………….. | p. 18 |
|  | Figure S6. Funnel Plot for Child Emotional Problems…………………………………………... | p. 19 |
|  | Figure S7. Funnel Plot for Parents’ Ineffective Parenting Practices…………………………….. | p. 20 |
|  | Figure S8. Funnel Plot for Parents’ Mental Health Problems…………………………………… | p. 21 |

Corresponding author: Ana Catarina Canário, Faculty of Psychology and Education Sciences of the University of Porto, Portugal; anacanario@fpce.up.pt

**Online Resource 1**

*PRISMA 27-item Checklist*

| **Section and Topic** | **Item #** | **Checklist item** | **Location where item is reported** |
| --- | --- | --- | --- |
| **TITLE** | | |  |
| Title | 1 | Identify the report as a systematic review. | M p.1 |
| **ABSTRACT** | | |  |
| Abstract | 2 | See the PRISMA 2020 for Abstracts checklist. | M p.1 |
| **INTRODUCTION** | | |  |
| Rationale | 3 | Describe the rationale for the review in the context of existing knowledge. | M pp.1-3 |
| Objectives | 4 | Provide an explicit statement of the objective(s) or question(s) the review addresses. | M p.3 |
| **METHODS** | | |  |
| Eligibility criteria | 5 | Specify the inclusion and exclusion criteria for the review and how studies were grouped for the syntheses. | M p.3 |
| Information sources | 6 | Specify all databases, registers, websites, organisations, reference lists and other sources searched or consulted to identify studies. Specify the date when each source was last searched or consulted. | M p.3 |
| Search strategy | 7 | Present the full search strategies for all databases, registers and websites, including any filters and limits used. | SM pp.4-5 |
| Selection process | 8 | Specify the methods used to decide whether a study met the inclusion criteria of the review, including how many reviewers screened each record and each report retrieved, whether they worked independently, and if applicable, details of automation tools used in the process. | M p.3 |
| Data collection process | 9 | Specify the methods used to collect data from reports, including how many reviewers collected data from each report, whether they worked independently, any processes for obtaining or confirming data from study investigators, and if applicable, details of automation tools used in the process. | M pp.3-4 |
| Data items | 10a | List and define all outcomes for which data were sought. Specify whether all results that were compatible with each outcome domain in each study were sought (e.g. for all measures, time points, analyses), and if not, the methods used to decide which results to collect. | M. pp.3-4 |
|  | 10b | List and define all other variables for which data were sought (e.g. participant and intervention characteristics, funding sources). Describe any assumptions made about any missing or unclear information. | M. pp.3-4 |
| Study risk of bias assessment | 11 | Specify the methods used to assess risk of bias in the included studies, including details of the tool(s) used, how many reviewers assessed each study and whether they worked independently, and if applicable, details of automation tools used in the process. | M p.4 |
| Effect measures | 12 | Specify for each outcome the effect measure(s) (e.g. risk ratio, mean difference) used in the synthesis or presentation of results. | M p.4 |
| Synthesis methods | 13a | Describe the processes used to decide which studies were eligible for each synthesis (e.g. tabulating the study intervention characteristics and comparing against the planned groups for each synthesis (item #5)). | M pp.3-4 |
|  | 13b | Describe any methods required to prepare the data for presentation or synthesis, such as handling of missing summary statistics, or data conversions. | M. p.4 |
|  | 13c | Describe any methods used to tabulate or visually display results of individual studies and syntheses. | M. pp.4-11 |
|  | 13d | Describe any methods used to synthesize results and provide a rationale for the choice(s). If meta-analysis was performed, describe the model(s), method(s) to identify the presence and extent of statistical heterogeneity, and software package(s) used. | M p.4 |
|  | 13e | Describe any methods used to explore possible causes of heterogeneity among study results (e.g. subgroup analysis, meta-regression). | M pp.10-13 |
|  | 13f | Describe any sensitivity analyses conducted to assess robustness of the synthesized results. | Not applicable |
| Reporting bias assessment | 14 | Describe any methods used to assess risk of bias due to missing results in a synthesis (arising from reporting biases). | M pp.13-14 |
| Certainty assessment | 15 | Describe any methods used to assess certainty (or confidence) in the body of evidence for an outcome. | Not applicable |
| **RESULTS** | | |  |
| Study selection | 16a | Describe the results of the search and selection process, from the number of records identified in the search to the number of studies included in the review, ideally using a flow diagram. | M pp.4-5 |
|  | 16b | Cite studies that might appear to meet the inclusion criteria, but which were excluded, and explain why they were excluded. | M Figure 1 |
| Study characteristics | 17 | Cite each included study and present its characteristics. | M Table 1 |
| Risk of bias in studies | 18 | Present assessments of risk of bias for each included study. | M pp.13-14  SM pp.16-17 |
| Results of individual studies | 19 | For all outcomes, present, for each study: (a) summary statistics for each group (where appropriate) and (b) an effect estimate and its precision (e.g. confidence/credible interval), ideally using structured tables or plots. | M pp.4-14 |
| Results of syntheses | 20a | For each synthesis, briefly summarise the characteristics and risk of bias among contributing studies. | M pp.4-14 |
|  | 20b | Present results of all statistical syntheses conducted. If meta-analysis was done, present for each the summary estimate and its precision (e.g. confidence/credible interval) and measures of statistical heterogeneity. If comparing groups, describe the direction of the effect. | M pp.10-13 |
|  | 20c | Present results of all investigations of possible causes of heterogeneity among study results. | M pp.10-13 |
|  | 20d | Present results of all sensitivity analyses conducted to assess the robustness of the synthesized results. | Not applicable |
| Reporting biases | 21 | Present assessments of risk of bias due to missing results (arising from reporting biases) for each synthesis assessed. | M pp.13-14  SM pp.16-21 |
| Certainty of evidence | 22 | Present assessments of certainty (or confidence) in the body of evidence for each outcome assessed. | Not applicable |
| **DISCUSSION** | | |  |
| Discussion | 23a | Provide a general interpretation of the results in the context of other evidence. | M pp.14-15 |
|  | 23b | Discuss any limitations of the evidence included in the review. | M pp.14-15 |
|  | 23c | Discuss any limitations of the review processes used. | M pp.14-15 |
|  | 23d | Discuss implications of the results for practice, policy, and future research. | M pp.14-15 |
| **OTHER INFORMATION** | | |  |
| Registration and protocol | 24a | Provide registration information for the review, including register name and registration number, or state that the review was not registered. | M p.3 |
|  | 24b | Indicate where the review protocol can be accessed, or state that a protocol was not prepared. | M p.3 |
|  | 24c | Describe and explain any amendments to information provided at registration or in the protocol. | Not applicable |
| Support | 25 | Describe sources of financial or non-financial support for the review, and the role of the funders or sponsors in the review. | M p.15 |
| Competing interests | 26 | Declare any competing interests of review authors. | M p.15 |
| Availability of data, code and other materials | 27 | Report which of the following are publicly available and where they can be found: template data collection forms; data extracted from included studies; data used for all analyses; analytic code; any other materials used in the review. | M p.15 |

*Note.* M = Manuscript; SM = Supplemental Material; p = page; pp = pages

**Online Resource 2**

*Search String Used in Each Database*

**PsycINFO**

*Ovid*

**#1 Parenting**

parent training/ OR parenting skills/ OR parent child relations/ OR father child relations/ OR mother child relations/ OR (((parent* OR mother* OR father* OR maternal) ADJ2 (training* OR intervention* OR program*)) OR family support program* OR parenting strateg* OR parental competenc* OR parent focused OR parent child interaction OR mother child interaction OR father child interaction OR parent child relations*).ti,ab,id.

**#2 Support**

intervention/ OR early intervention/ OR family intervention/ OR (training* OR intervention* OR program* OR educa* OR therap* OR treatment* OR coach* OR counseling).ti,ab,id.

**#3 Online**

online therapy/ OR social media/ OR blog/ OR internet/ OR online community/ OR websites/ OR (Internet* OR computer* OR online OR on-line OR email* OR mail* OR chat* OR web* OR social media OR twitter OR facebook OR youtube OR snapchat OR instagram OR telegram OR e-health OR blog* OR digital OR social network* OR whatsapp).ti,ab,id.

**1 AND 2 AND 3**

**Medline**

*Ovid*

**#1 Parenting**

parent-child relations/ or father-child relations/ or mother-child relations/ OR (((parent* OR mother* OR father* OR maternal) ADJ2 (training* OR intervention* OR program*)) OR family support program* OR parenting strateg* OR parental competenc* OR parent focused OR parent child interaction OR mother child interaction OR father child interaction OR parent child relations*).ti,ab,kf.

**#2 Support**

(training* OR intervention* OR program* OR educa* OR therap* OR treatment* OR coach* OR counseling).ti,ab,kf.

**#3 Online**

blogging/ OR internet/ OR social media/ OR social networking/ OR Therapy, Computer-Assisted/ OR telemedicine/ OR (Internet* OR computer* OR online OR on-line OR email* OR mail* OR chat* OR web* OR social media OR twitter OR facebook OR youtube OR snapchat OR instagram OR blog* OR digital OR social network* OR whatsapp OR telegram OR e-health).ti,ab,kf.

**1 AND 2 AND 3**

**Web of Science**

**#1 Parenting**

TS=((("parent*" OR "mother*" OR "father*" OR "maternal") NEAR/1 ("training*" OR "intervention*" OR "program*")) OR "family support program*" OR "parenting strateg*" OR "parental competenc*" OR "parent focused" OR "parent child interaction" OR "mother child interaction" OR "father child interaction" OR "parent child relations*")

**#2 Support**

TS=("training*" OR "intervention*" OR "program*" OR "educa*" OR "therap*" OR "treatment*" OR "coach*" OR "counseling")

**#3 Online**

TS=("internet*" OR "computer*" OR "online" OR "on-line" OR "email*" OR "mail*" OR "chat*" OR "web*" OR "social media" OR "twitter" OR "facebook" OR "youtube" OR "snapchat" OR "instagram" OR "blog*" OR "digital" OR "social network*" OR "whatsapp" OR "telegram" OR "e-health")

**1 AND 2 AND 3**

**Cochrane Library**

**#1 Parenting**

(("parent*":ti,ab,kw OR "mother*":ti,ab,kw OR "father*":ti,ab,kw OR "maternal":ti,ab,kw) NEAR/1 ("training*":ti,ab,kw OR "intervention*":ti,ab,kw OR "program*":ti,ab,kw)) OR "family support program*":ti,ab,kw OR "parenting strateg*":ti,ab,kw OR "parental competenc*":ti,ab,kw OR "parent focused":ti,ab,kw OR "parent child interaction":ti,ab,kw OR "mother child interaction":ti,ab,kw OR "father child interaction":ti,ab,kw OR "parent child relations*":ti,ab,kw

**#2 Support**

"training*":ti,ab,kw OR "intervention*":ti,ab,kw OR "program*":ti,ab,kw OR "educa*":ti,ab,kw OR "therap*":ti,ab,kw OR "treatment*":ti,ab,kw OR "coach*":ti,ab,kw OR "counseling":ti,ab,kw

**#3 Online**

"internet*":ti,ab,kw OR "computer*":ti,ab,kw OR "online":ti,ab,kw OR "on-line":ti,ab,kw OR "email*":ti,ab,kw OR "mail*":ti,ab,kw OR "chat*":ti,ab,kw OR "web*":ti,ab,kw OR "social media":ti,ab,kw OR "twitter":ti,ab,kw OR "facebook":ti,ab,kw OR "youtube":ti,ab,kw OR "snapchat":ti,ab,kw OR "instagram":ti,ab,kw OR "blog*":ti,ab,kw OR "digital":ti,ab,kw OR "social network*":ti,ab,kw OR "whatsapp":ti,ab,kw OR "telegram":ti,ab,kw OR "e-health":ti,ab,kw

**1 AND 2 AND 3**

**Online Resource 3**

**Table S1**

*Online Parenting Programs’ Components: Theoretical Approaches and Contents*

| **Theoretical orientation** | **Content** |
| --- | --- |
| Psychoeducation  Underlying rationale: “Providing knowledge about typical and atypical child development and parent-child interactions will enhance adaptive parenting practices (e.g., less harshness and hostility)” | **Knowledge transfer** contents, i.e., parents are informed about general child development and parent-child interactions. |
| Relationship perspectives  Underlying rationale: “Strengthening the parent-child relationship satisfies children’s need for relatedness increases reciprocity, and will enhance adaptive parenting practices (e.g., less harshness and hostility)” | **Positive activities / positive involvement** contents, where parents learn to engage in positive, fun activities (e.g., play) and/or be involved in the child’s activities (e.g., homework) |
|  | **Child-led activities** contents, where parents learn to engage in activities where the child takes the lead, to increase parental awareness of the child’s needs and meet children’s need for autonomy |
|  | **Mind-mindedness or empathy** contents, where parents learn to understand the child’s mind and feelings. |
| Learning theory perspectives  Underlying rationale: “behavior that is rewarded will increase; behavior that is not rewarded will decrease” | **Positive reinforcement** contents, where parents learn to react to positive child behavior with praise and/or rewards. |
|  | **Non-violent disciplining techniques** contents, where parents learn to react to disruptive child behavior with a nonviolent consequence that is intended to reduce the behavior (time-out, ignore, and/or natural or logical consequences). |
| Proactive parenting  Underlying rationale: “Parent-child interactions can be structured such that disruptive behavior is unlikely to occur” | **Direct commands** contents, where parents learn to proactively give children direct and positive commands (e.g., instruct rather than ask or beg, and tell children to “do” something rather than “not to do” something). |
|  | **Clear limits** contents, where parents learn to set rules about appropriate and inappropriate behavior. |
|  | **Monitoring** contents, where parents learn to invest in knowing what the child does and whom they play with. |
| **Theoretical orientation** | **Content** |
| Parental self-care  Underlying rationale: “when parents take good care of themselves, this will enhance adaptive parenting (e.g., less harshness and hostility)” | **Parental stress reduction** contents, where parents learn to engage in activities that reduce parental stress (e.g., mindfulness). |
|  | **Parental emotion regulation** contents, where parents learn to recognize and regulate your own feelings as a parent (e.g., counting till ten before responding). |
|  | **Parental problem-solving skills** contents, where parents learn to generate and implement solutions to difficult parenting situations. |
|  | **Parental partner support** contents, where parents learn to invest improve partner relationships and co-parenting. |
| Parents as therapist  Underlying rationale: “parents can teach their children skills that will enhance their mental health” | **Child emotion regulation skills** contents, where parents learn about teaching the child how to have words for emotions and how to regulate them.  **Child problem-solving skills** contents, where parents learn about teaching the child how to solve everyday problems.  **Child social skills** contents, where parents learn about teaching the child how to interact with other children.  **Exposure** contents, where parents expose the child to feared stimuli.  **Avoidance** contents, where parents manage children's anxiety-driven avoidance behaviors. |

**Online Resource 4**

*Scripts Used to Performed Network Meta-Analysis to Compare the Effects of Each Cluster of Components on Child Behavioral Problems*

# Load metafor package

install.packages("metafor")

library("metafor")

# Convert data in escalc object

dat <- escalc(measure="SMD", yi=CohensD, vi=varcohensd, data=dat)

#aggregate (creates new data set with aggregate data behav problems aggregate)

bpagg <- aggregate(dat, cluster=Compid, rho=0.5)

#generate SE

library(dplyr)

bpagg <- mutate(bpagg, dse=sqrt(varcohensd))

#install and load network meta analysis package

install.packages("netmeta")

library(netmeta)

bpnma <- netmeta(TE=CohensD, seTE=dse, treat1=Treatcode1A, treat2=Treatcode2, reference.group="CONTROL",

studlab=Trial, random=TRUE, method.tau="REML", data=bpagg)

# summary

bpcomb <- netcomb(bpnma, inactive="CONTROL")

summary(bpcomb)

netgraph(bpcomb)

forest(bpcomb)

netrank(bpcomb)

*Scripts Used to Performed Network Meta-Analysis to Compare the Effects of Each Cluster of Components on Child Emotional Problems*

# Load metafor package

install.packages("metafor")

library("metafor")

# Convert data in escalc object

dat <- escalc(measure="SMD", yi=CohensD, vi=varcohensd, data=dat)

#aggregate (creates new data set with aggregate data emotional problems aggregate)

epagg <- aggregate(dat, cluster=Compid, rho=0.5)

#generate SE

library(dplyr)

epagg <- mutate(epagg, dse=sqrt(varcohensd))

#install and load network meta analysis package

install.packages("netmeta")

library(netmeta)

epnma <- netmeta(TE=CohensD, seTE=dse, treat1=Treatcode1A, treat2=Treatcode2, reference.group="CONTROL",

studlab=Trial, random=TRUE, method.tau="REML", data=epagg)

# summary

epcomb <- netcomb(epnma, inactive="CONTROL")

summary(epcomb)

netgraph(epcomb)

forest(epcomb)

netrank(epcomb)

**Online Resource 5**

**Table S2**

*Components and Respective Theoretical Approach Identified in Each Online Parenting Program*

| Online program | Components and respective theoretical orientation | | | | | | | | | | | | | | | | | | |
| --- | --- | --- | --- | --- | --- | --- | --- | --- | --- | --- | --- | --- | --- | --- | --- | --- | --- | --- | --- |
|  | KT (PE) | PA (RP) | CLA  (RP) | ME  (RP) | PR  (LTp) | NVD  (LTp) | DC  (PP) | CL  (PP) | M  (PP) | PSR  (PSC) | PER  (PSC) | PPS  (PSC) | PS  (PSC) | CER  (PT) | CPS  (PT) | CSS  (PT) | E  (PT) | A  (PT) |  |
| Adventures in Parenting | - |  | - |  |  |  |  |  |  | - |  |  | - |  |  | - | - | - |  |
| Child Anxiety Tales |  | - | - | - |  |  | - | - | - |  |  |  | - |  | - | - |  |  |  |
| Children in Between |  | - | - | - | - | - |  | - | - | - | - |  |  | - | - | - | - | - |  |
| Confident Parent Internet Guide program |  | - | - | - |  |  | - | - | - | - | - | - | - | - | - | - | - | - |  |
| Cool Little Kids Online |  | - |  | - |  | - | - | - | - | - |  |  | - |  | - | - |  |  |  |
| EMPOWER |  | - | - | - | - | - | - | - | - | - | - | - | - |  | - | - |  |  |  |
| ezParent Program | - |  | - | - |  |  | - | - | - |  |  |  | - | - | - | - | - | - |  |
| Fathering Through Change |  |  | - |  |  |  |  | - | - | - |  |  | - |  |  | - | - | - |  |
| Group Executive Functioning and Online Parent Training |  |  | - | - |  |  |  | - | - |  | - | - | - | - | - | - | - | - |  |
| iCALM Telehealth Program |  |  | - | - |  |  |  | - | - | - | - |  | - |  | - | - |  |  |  |
| Internet-based Parent Management Treatment | - |  | - | - |  |  | - | - | - | - | - | - | - | - | - | - | - | - |  |
| Modified version of the BRAVE-ONLINE |  | - | - | - | - | - | - | - | - |  | - | - | - | - |  |  |  |  |  |
| Online Behavioral Parent Training | - |  | - | - |  | - | - | - | - | - | - |  | - | - | - | - | - | - |  |
| Online Mindful Parenting Training |  | - | - |  | - | - | - | - | - |  | - |  | - | - | - | - | - | - |  |
| Online Positive Parenting Programme |  |  | - |  |  |  | - |  | - |  |  | - | - | - | - | - | - | - |  |
| Parent Positive App |  |  | - | - |  |  | - | - | - |  | - | - | - |  | - | - | - | - |  |
| Parenting Resilient Kids |  | - |  |  | - | - | - |  |  | - | - |  | - |  |  |  | - | - |  |
| Self-Help Parenting Program |  |  | - | - |  |  |  |  | - | - | - | - | - | - |  | - | - | - |  |
| Strongest Families Smart Website | - | - | - | - |  |  | - | - | - | - |  |  | - |  | - |  | - | - |  |
| Triple P Online |  |  | - | - |  |  |  |  |  |  |  |  |  |  |  |  | - | - |  |
| Triple P Online Brief |  |  | - | - |  |  |  |  |  | - | - |  | - |  | - |  | - | - |  |
| Turtle Program |  | - |  | - | - | - | - | - | - | - | - | - | - | - | - | - |  | - |  |
| Two Families Now | - | - | - | - | - | - | - | - | - |  | - |  |  | - | - | - | - | - |  |

*Note.* KT = Knowledge Transfer; PA = Invest in Positive Activities/Positive Involvement; CLA = Child-Led Activities; ME = Mind-Mindedness or Empathy; PR = Positive Reinforcement; NVD = Non-Violent Disciplining Techniques; DC = Direct Commands; CL = Clear Limits; M = Monitoring; PSR = Parental Stress Reduction; PER = Parental Emotion Regulation; PPS = Parental Problem-Solving Skills; PS = Parental Partner Support; CER = Child Emotion Regulation Skills; CPS = Child Problem-Solving Skills; CSS = Child Social Skills; E = Exposure; A = Avoidance; PE = Psychoeducation; RP = Relationship Perspectives; LTp = Learning Theory perspectives; PP = Proactive Parenting; PSC = Parental Self-Care; PT = Parents as Therapist.


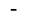

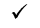
= Component identified in the online parenting program

= Component not identified in the online parenting program

**Online Resource 6**

**
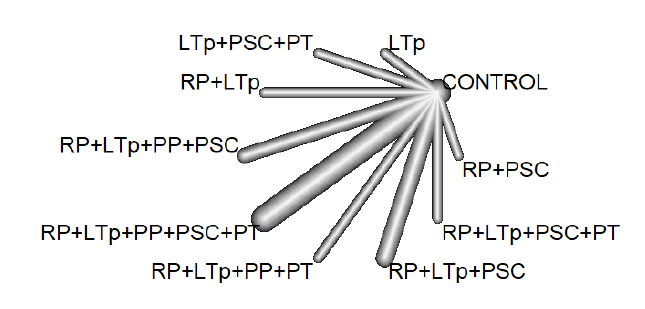
**

**Figure S1**

*Network Plot of Eligible Comparisons of Clusters of Components for Child Behavioral Problems*

*Note*. LTp = Learning Theory perspectives; PSC = Parental Self-Care; PT = Parents as Therapist; RP = Relationship Perspectives; PP = Proactive Parenting; CONTROL = Control condition (no or minimal intervention); The thickness of the lines indicates the number of trials per cluster

**Online Resource 7**

**Table S3**

*Ranking of Clusters of Components from the Cluster Most Likely to be Most Effective to the Cluster Least Likely to be Most Effective in Reducing Children’s Behavioral Problems*

| Cluster of components | P-score (common) |
| --- | --- |
| LTp+PSC+PT | 1.000 |
| LTp | 0.889 |
| RP+LTp+PSC+PT | 0.709 |
| RP+LTp+PP+PSC+PT | 0.724 |
| RP+LTp+PSC | 0.490 |
| RP+LTp+PP+PT | 0.260 |
| RP+LTp+PP+PSC | 0.505 |
| Control | 0.309 |
| RP+LTp | 0.114 |
| RP+PSC | 0.000 |

*Note*. LTp = Learning Theory perspectives; PSC = Parental Self-Care; PT = Parents as Therapist; RP = Relationship Perspectives; PP = Proactive Parenting; P-score = Position of clusters of components relative to others and certainty of that position provided by ranking metrics

**Online Resource 8**

**
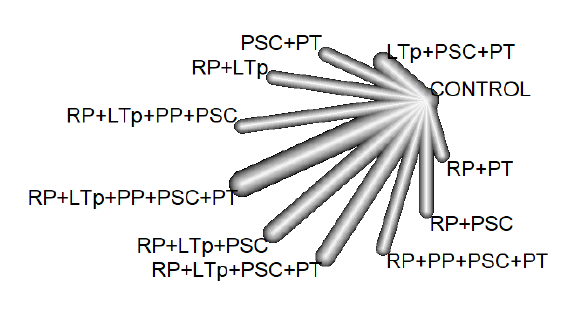
**

**Figure S2**

*Network Plot of Eligible Comparisons of Clusters of Components for Child Emotional Problems*

*Note*. LTp = Learning Theory perspectives; PSC = Parental Self-Care; PT = Parents as Therapist; RP = Relationship Perspectives; PP = Proactive Parenting; CONTROL = Control condition (no or minimal intervention); The thickness of the lines indicates the number of trials per cluster

**Online Resource 9**

**Table S4**

*Ranking of Clusters of Components from the Cluster Most Likely to be Most Effective to the Cluster Least Likely to be Most Effective in Reducing Children’s Emotional Problems*

| Cluster of components | P-score (common) |
| --- | --- |
| LTp+PSC+PT | 1.000 |
| RP+LTp+PSC+PT | 0.799 |
| PSC+PT | 0.900 |
| RP+LTp+PP+PSC+PT | 0.707 |
| RP+LTp+PSC | 0.554 |
| RP+LTp | 0.131 |
| RP+PT | 0.003 |
| Control | 0.517 |
| RP+LTp+PP+PSC | 0.431 |
| RP+PP+PSC+PT | 0.295 |
| RP+PSC | 0.173 |

*Note*. LTp = Learning Theory perspectives; PSC = Parental Self-Care; PT = Parents as Therapist; RP = Relationship Perspectives; PP = Proactive Parenting; P-score = Position of clusters of components relative to others and certainty of that position provided by ranking metrics

**Online Resource 10**

*
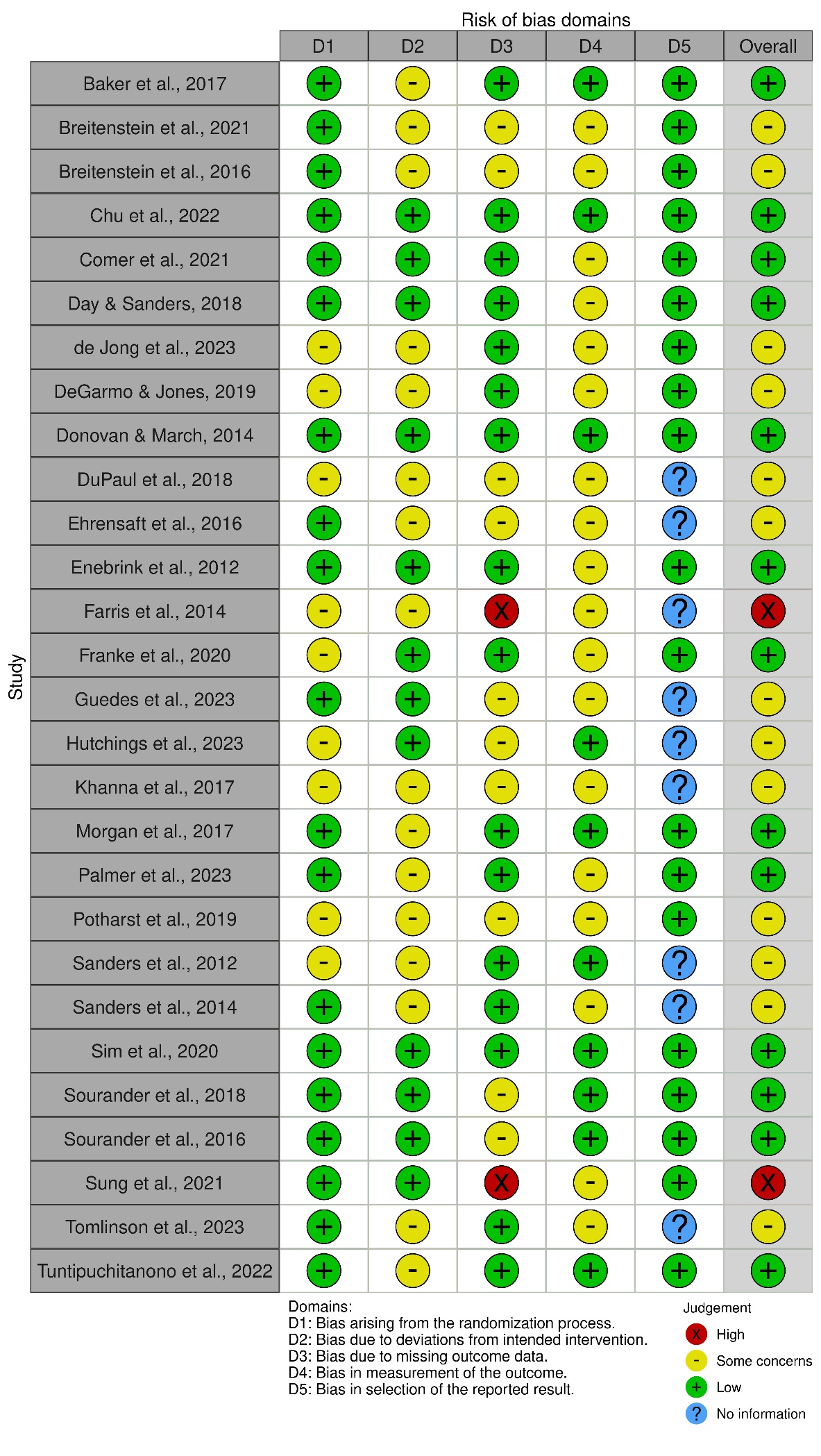
*

**Figure S3**

*Risk of Bias Traffic Light Plot at the Study Level Depicting Review Authors' Judgments about Each Risk of Bias*

*
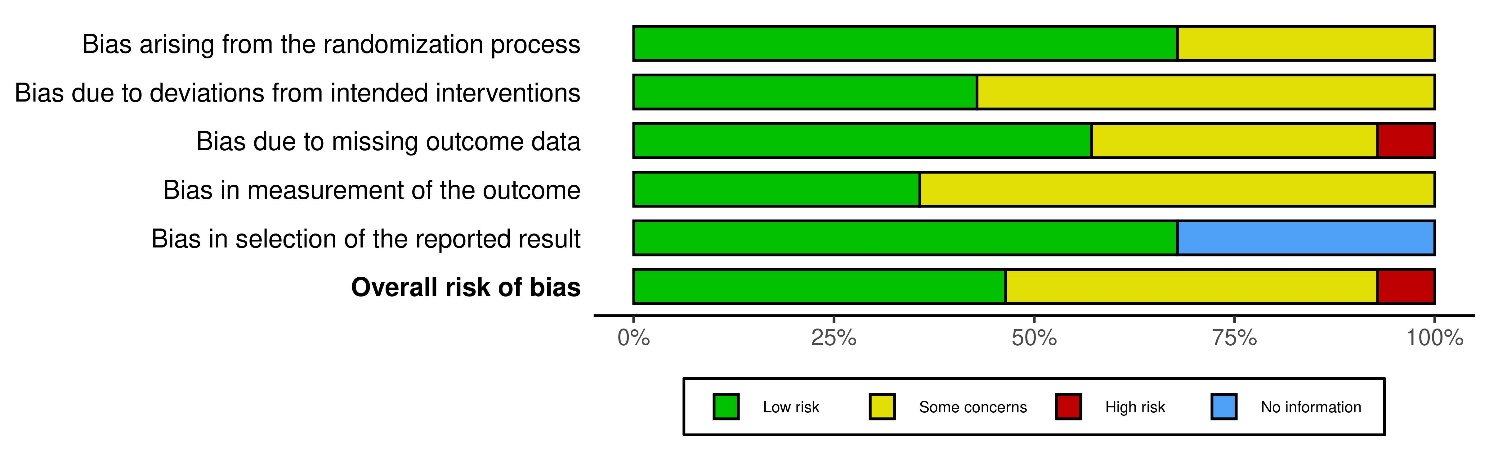
*

**Figure S4**

*Risk of Bias Summary Plot Depicting Review Authors' Judgments About Each Risk of Bias Item Presented as Percentages across all Included Studies*

**Online Resource 11**


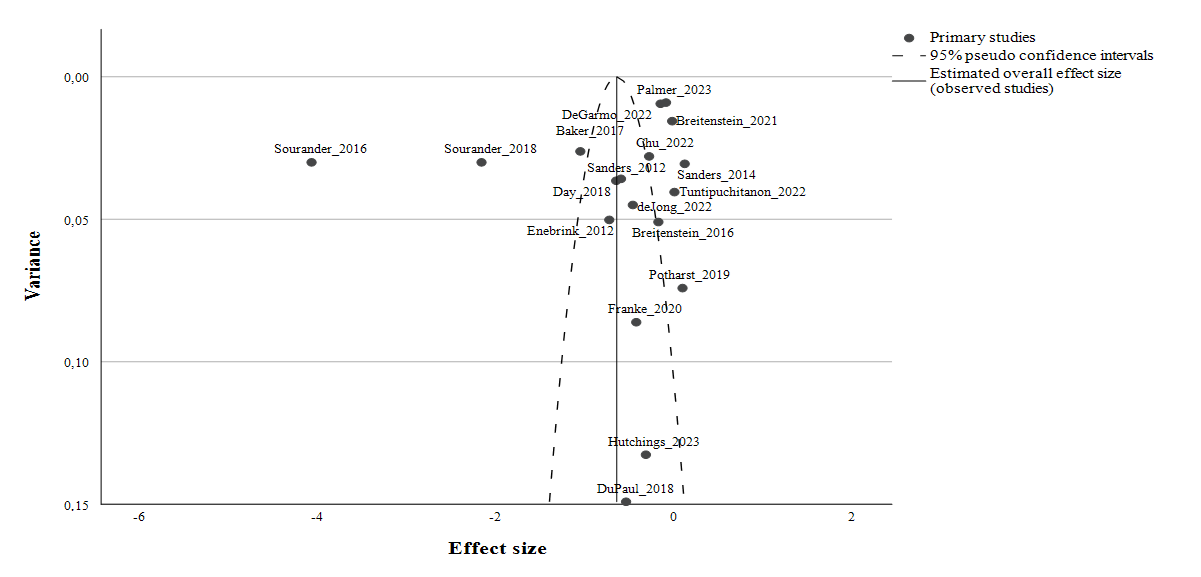


**Figure S5**

*Funnel Plot for Child Behavioral Problems*

**
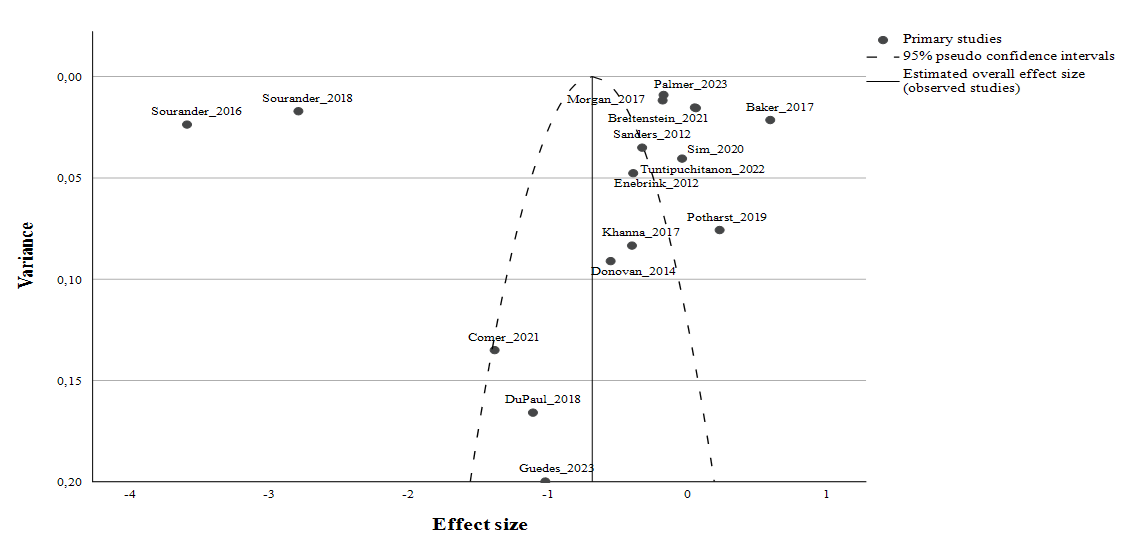
**

**Figure S6**

*Funnel Plot for Child Emotional Problems*

**
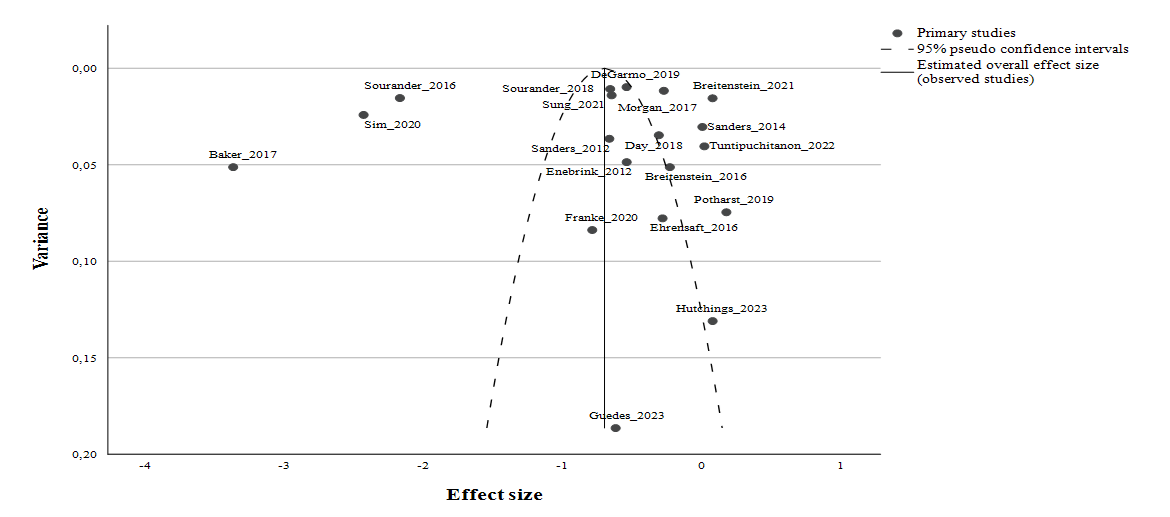
**

**Figure S7**

*Funnel Plot for Parents’ Ineffective Parenting Practices*

**
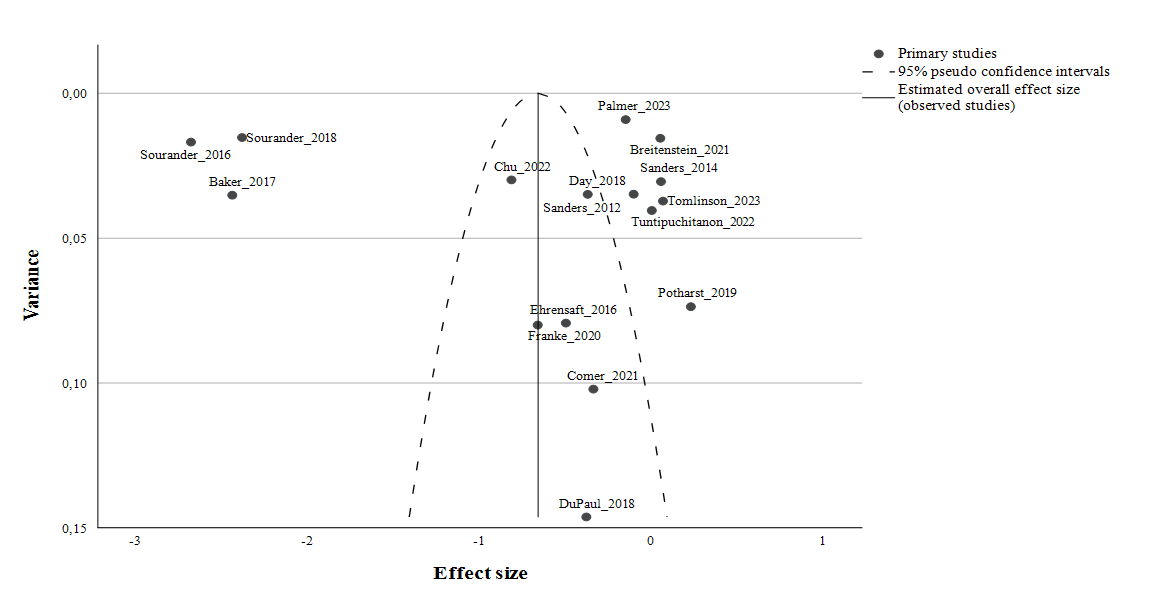
**

**Figure S8**

*Funnel Plot for Parents’ Mental Health Problems*
